# Targeting MERTK tyrosine kinase: Virtual screening and molecular dynamics insights for anti-cancer drug development

**DOI:** 10.1371/journal.pone.0334106

**Published:** 2025-10-30

**Authors:** Kashif Kifayat, Kamaljot Singh, Mahmood Khan, Dur E. Maknoon Razia, Sara Khan, Chengyong Dong, Liming Wang

**Affiliations:** 1 Division of Hepatobiliary and Pancreatic Surgery, Department of General Surgery, The Second Affiliated Hospital of Dalian Medical University, Dalian, China; 2 Department of Chemistry, Faculty of the Basic and Applied Sciences, Sri Guru Granth Sahib World University, Fatehgarh Sahib, Punjab, India; 3 Key Laboratory of Biorheological Science and Technology of Ministry of Education, College of Bioengineering, Chongqing University, Chongqing, China; 4 Department of Pathogen Biology, College of Basic Medicinal sciences, Jilin University, Changchun, China; 5 Department of Chemistry, COMSATS University Islamabad, Abbottabad Campus, Khyber Pakhtunkhwa, Pakistan; University of Colorado - Anschutz Medical Campus, UNITED STATES OF AMERICA

## Abstract

Global public health facing serious challenges due to the incidence of cancer and the growth of treatment resistance. Mer-tyrosine kinase plays a crucial role in cell biology and correlated with many cancers such as Epithelial ovarian cancer, liver cancer, breast cancer, Metastatic melanoma, and Acute myeloid leukemia (AML). Hence the identification of novel drug for MERTK protein is extreme important. In this research, we used computational techniques, molecular operating environment (MOE 2015) for virtual screening with drug like natural compounds library. We used known compound UNC2025 as positive control and one million compounds was retrieved from different databases (OTAVA, ZINC, ChEMBL) and docked with MERTK protein. Out of million compounds the 4 top hit inhibitors chosen from docking were further screened for ADMET profiling confirming their compliance with drug designing and toxicological principle and subjected to molecular dynamic (MD) simulation and MM-PBSA analysis. The results of these analyses showed that only four compounds that make strong interactions with MERTK protein *via* highest binding affinity hydrogen bond and hydrophobic contacts (lig1, lig2, lig3, lig4). The computed binding affinity ranges from –22.977 to –18.707 kcal/mol. The increased helix and reduced β–sheet contents in MERTK on the binding of top hit candidates depicted the higher structural stability of MERTK, rather than MERTK alone and MERTK–UNC2025. The study finds critical residues which serve a vital part in binding with the inhibitor and the active site of the MERTK protein, *i.e.*, Phe598, Gly599, Lys619, Arg629, Glu633, Glu637, Arg722, Asp723, Arg727, Asp741, Gly743, Leu744, Lys746, Arg758, Ala760, and Lys761 through decomposed binding free energy analysis. This study focuses on the pursuit of several MERTK protein targets, which could have consequences for the development of novel therapeutics for various cancers.

## 1. Introduction

Myeloid-epithelial-reproductive tyrosine kinase (MERTK) is a tyrosine kinase receptor of the tumor-associated macrophage (TAM) family. MERTK is a transmembrane protein that has a predominant expression in hematopoietic heredities, comprising natural killer (NK) cells, dendritic cells (DCs), monocytes, and macrophages [1]. The structure of MERTK comprises of extracellular domain with two immunoglobulin-like domains and two FNIII domains along with an intracellular tyrosine kinase domain [2]. The main ligand of (TAMs) consists of protein S (ProsI) and Growth arrest specific factor 6 (Gas6), which are comparable protein structure that needs vitamin K (vit-k) depended γ- carboxylation in instruction to activate the tyrosine kinase activities associated with MERTK activation [3]. Gas6 and Pros1 indicate approximately 44% homology in their amino acid sequences. Also, both proteins possess similar domain structures, which include an N-terminal γ-carboxyl-glutamic acid (Glu) domain, four consecutive Epidermal Growth Factor (EGF)-like repeats, and a C-terminal region known as Sex Hormone-Binding Globulin-like (SHBG), which comprises two Laminin G (LG) repeats [4,5]. One of MERTK’s roles is to aid in the phagocytosis process, which involves the efficient engulfment and clearance of apoptotic cells, allowing their identification and elimination from the body [6].

MERTK has critical roles through developmental, physiological, and pathological processes. Moreover, MERTK plays a key regulator of endothelial barrier function by inhibiting neutrophil trans endothelial migration (TEM) and endothelial cell (EC) permeability. This regulatory activity is essential for preserving proper barrier function under both homeostatic and inflammatory conditions. It is important to note that the understanding of MERTK’s functions is still evolving, and ongoing research continues to uncover its roles in various physiological and pathological processes. The diverse functions of MERTK highlight its significance in maintaining cellular and tissue homeostasis throughout the body [7]. Mutations in this gene have been associated with the alteration of the retinal pigment epithelium (RPE) phagocytosis pathway, causing the initial stages of autosomal recessive retinitis pigmentosa (RP) [8]. Activation of MERTK, similar to many receptor tyrosine kinases (RTKs), initiates typical RTK post-receptor signaling pathways. This involves the activation of ERK1/2, Akt, YAP/TAZ, and p38 MAP kinases, as well as Janus-activated kinase (JAK)/STAT, FAK/RhoA/MLC2, and Bcl-2 family members, which all contribute to activities such as cell invasion, migration, angiogenesis, cell survival, chemoresistance, and metastasis [9]. The MERTK kinase domain exhibits the canonical bilobal architecture characteristic of protein kinases, consisting of an N-terminal lobe (residues 578–670) and a larger C-terminal lobe (residues 680–861), connected by a hinge segment (residues 672–679) containing the gatekeeper residue Leu671 [10]. The ATP-binding cleft is situated at the interface of the two lobes, defined by the hinge region and the glycine-rich loop (G-loop; residues 591–601), which together facilitate nucleotide binding and orientation for catalysis [11].

Dysregulation of MERTK has been observed in various types of cancers, implicating its involvement in tumorigenesis and cancer progression. Here are some instances of MERTK dysregulation in different cancers, like increased expression of MERTK has been associated with breast cancer progression. It may promote tumor development and metastasis. Furthermore, in a significant proportion of primary breast carcinomas from patients experiencing relapse, the diminished expression of miR-335—an identified inhibitor of cancer metastasis calculated to target the 3’-UTR of MERTK possibly explains the Upregulation of MERTK in these susceptible tumors [12]. Epithelial ovarian cancer (EOC) poses a significant medical challenge, causing over 100,000 annual deaths in Western countries according to the TCGA patient dataset. The genes MMP9, FOXP1, DLL4, CD44, MERTK, and PTPRC were observed to have higher expression levels in tumors that recurred following CIS platinum treatment compared to untreated tumors. MERTK overexpression has been reported in ovarian cancer, and its dysregulation is connected to inadequate prognosis and resistance to chemotherapy [13]. Gastric cancer (GC) has significant heterogeneity, and there is evidence of increased expression of MERTK in gastric cancer. The observed increase in elevation suggests a potential role of MERTK in promoting tumor proliferation and metastasis. According to recent research, it was shown that 8.3% (16 out of 192) of patients diagnosed with gastric cancer had a significantly elevated level of total MERTK protein expression. Moreover, these patients exhibited overall negative survival outcomes. Furthermore, findings demonstrated a significant inhibition of MERTK-overexpressing GC cells following MERTK knockdown, indicating MERTK might be a possible novel therapeutic target in the treatment of gastric cancer [14]. Due to its aggressiveness, metastatic melanoma is one of the deadliest types of cutaneous cancers. MERTK, which has been identified for its oncogenic characteristics, is commonly overexpressed or activated in many cancers. We demonstrate the correlation between MERTK expression and disease progression using a combination of protein immunohistochemistry and microarray analysis. Notably, the expression of MERTK was maximum in metastatic melanomas, subsequent to primary melanomas, and lowest in nevi. Furthermore, when melanoma cell lines were associated to usual human melanocytes, almost half of them showed MERTK overexpression [15]. Acute myeloid leukemia (AML) still has a poor prognosis, with survival rates of less than 50% in adults and only 60% to 70% in children, despite recent treatment advances [16]. The majority of acute leukemias have aberrant expression of the receptor tyrosine kinase MERTK, and new data suggest that it may also be involved in a number of solid malignancies. In comparison to normal bone marrow precursor cells, it is shown that MERTK is overexpressed in more than 80% of pediatric and adult AML patient samples. In AML, blocking MERTK with shRNA reduced signaling *via* prosurvival pathways, limited colony formation, caused apoptosis, and boosted survival in animal models. This underscores how MERTK inhibition can be utilized therapeutically for AML [17,18]. Ongoing research continues to unveil the multifaceted roles of MERTK in physiological and pathological processes. The diverse functions of MERTK underscore its significance in maintaining cellular and tissue homeostasis throughout the body. The identified dysregulations in various cancers highlight MERTK as a potential target for novel therapeutic interventions.

Recent advances have underscored MERTK as a promising therapeutic target owing to its role in tumor cell survival, immune evasion, and macrophage-mediated immunosuppression [19–21]. Structure-based efforts have yielded novel macrocyclic pyrrolopyrimidines with nanomolar potency and *in vivo* efficacy (UNC3133), as well as highly selective dual MERTK/AXL inhibitors based on a 2-amino-3-carboxamidepyridine moiety, such as A-910 with favorable pharmacokinetic profiles [22,23]. Selectivity has also been enhanced by exploiting conformational dynamics of the αC helix, leading to inhibitors like compound 11 (Furo-[2,3-*d*]pyrimidine-based derivative) with strong MERTK specificity [24]. Beyond kinase inhibition, MERTK-expressing macrophages have been linked to tumor progression and resistance to immunotherapy, and targeting MERTK signaling in macrophages has been shown to improve responses to immune checkpoint blockade [25]. Complementary to medicinal chemistry approaches, computational strategies such as reaction-based *de novo* design and scaffold enrichment have identified a novel MERTK inhibitor, compound 5a (IC_50_ = 53.4 nM) [26]. Dual inhibition strategies have also emerged, including UNC9435, the first TYRO3/MERTK dual inhibitor [27] and macrocyclic MERTK/AXL inhibitors with promising activity in non-small cell lung cancer [28]. Pyrazinamide-based inhibitors (compound 31: IC_50_ = 1.3 nM) and bifunctional scaffolds such as BPR5K230 have further demonstrated both direct antitumor activity and immune-modulatory properties [29,30]. Collectively, these studies highlight the therapeutic promise of MERTK inhibition and the diversity of approaches ranging from macrocyclic and bifunctional inhibitors to AI-assisted design.

While most of the literature studies have only focused on experimental [19–21] discovery of small subsets of compounds, only a few studies have focused on computational [31,32] evaluation of inhibitors against MERTK. Thus, in the present work, computational techniques were used to find novel drug candidates by promoting the efficiency of drug discovery as *in silico* screening plays a key role in identifying hit compounds from a large databases, thereby advancing drug development [33]. In this regard, virtual screening of 1 million compounds from the natural product-like library available in the OTAVA, ZINC and ChEMBL databases [34–36] was performed in combination with molecular docking and molecular dynamics simulations, using the clinically relevant inhibitor UNC2025 as a positive control. Zhang et al. developed UNC2025 as a potent, pharmacologically selective MERTK inhibitor (IC_50_ = 0.74 nM), exhibiting oral bioavailability, improved solubility, and favorable DMPK (drug metabolism and pharmacokinetics) properties [37]. Further, UNC2025 strongly suppressed prosurvival signaling, triggered apoptosis, and markedly reduced proliferation and colony formation in MERTK-expressing acute lymphoblastic leukemias (ALL), acute myeloid leukemias (AML), melanoma cell lines, as well as patient-derived samples in preclinical trials [38,39]. The comparative framework of top hits with UNC2025 not only validates the employed computational strategy but also provides mechanistic insights into binding interactions and stability of potential MERTK inhibitors.

## 2. Methodology

### 2.1. Protein preparation

The RCSB protein Data Bank provided the 3D structure of crystalized of MERTK (PDB ID: 7XHY) to a known potent selective inhibitor [40]. Maestro 12.8 wizard tool was utilized to prepare the 3D structure of MERTK protein [41]. This step included the addition of hydrogen atoms, assigning bond orders to essential amino acid residues, eliminating heteroatoms and solvent molecules that exceeded 5, optimizing hydrogen bonds, and generating zero-order bonds with metals.

In addition, the eliminated side chains and loops were added using a Maestro Prime module option. Following that, the energy of the preprocessed and optimized MERTK structure was reduced using the OPLS force field [42,43].

### 2.2. Ligand preparation

PubChem was used to obtain the known compound UNC2025 for analysis, and 1 million natural compounds were retrieved from OTAVA, ZINC and ChEMBL databases in mol2 format [34–36]. The protonate 3D model of MOE software was used to introduce partial charges to compounds [44]. Afterwards, the energies of ligands were reduced by employing the MMFF94X force field.

### 2.3. Molecular docking

In the computational technique, drug designing is productive and cost-effective in molecular docking to classify and evaluate molecular interactions between the ligand and receptors [45]. In the current research, the Genetic Optimization for Ligand Docking method was utilized to dock the previously mentioned drug-like database made from natural sources with the active site of MERTK protein, site finder tool was used to identify the active site of the protein. MOE software was used for docking with known and natural compounds against MERTK with an identified docking site [44]. The binding energies were computed using Generalized-Born solvation models, while the receptor amino acids were kept rigid. Based on the binding energy, root-mean-square deviation (RMSD), and S-score function, we selected six complexes for further research.

### 2.4. Ligand–receptor interaction analyses

The interaction analyses of docked complexes were performed using the MOE’s ligX program to generate 2D plots of receptor ligand interactions that highlighted hydrogen bonding, hydrophobic interactions, and electrostatic/non-electrostatic interactions [44]. The above interactions demonstrated that compounds bind to a protein’s active site. The PyMOL software was used to display the 3D structure of complexes [46]. The five compounds were selected from docking with highest docking score, including UNC2025 (reference) for MD simulation [37].

### 2.5. Drug-Likeness evaluation

The SwissADME tool (http://www.swissadme.ch/index.php) was utilized to predict the drug likeness properties of compounds [47].

### 2.6. Protocol for Molecular Dynamics (MD) simulations and analysis

The MD simulations of the optimal docked MERTK-ligand complexes were conducted for 200 ns employing GROMACS-2022.4 [48]. A total of six systems [MERTK (alone), MERTK–UNC2025, MERTK–lig1, MERTK–lig2, MERTK–lig3, and MERTK–lig4] were meticulously prepared utilizing the all-atom AMBER99SB-ILDN force field [49], with TIP3P water molecules to solvate the systems, ensuring a distance of 1.0 nm from the periodic edges of the box. The Automated Topology Builder and Repository (ATB) [50] was used to generate the parameters of the AMBER99SB-ILDN force field [49] for ligands in topology format. The protocol for further system preparation followed the methodology outlined in our previous study [51]. The energy minimization of each system was performed using the steepest descent algorithm to relieve steric clashes. Further, the systems were equilibrated in two stages: first under the NVT ensemble for 1000 ps to stabilize the system at 310 K, followed by the NPT ensemble for 1000 ps at 1 bar to equilibrate pressure and density. All bond lengths involving hydrogen atoms were constrained using the LINCS algorithm [52], while the SETTLE algorithm [53] was employed to constrain the bond length within water molecules. Long-range electrostatics were calculated using the Particle Mesh Ewald (PME) method, while a cutoff of 1.2 nm was applied for short-range van der Waals interactions [54,55]. The temperature was controlled using the Nosé–Hoover thermostat [56], and pressure was maintained with the Parrinello–Rahman barostat [57] during the production of 200 ns MD simulations for each system. Afterwards, each system underwent a 200 ns run of MD simulations production, which were analyzed and visualized with GROMACS utilities [48] and PyMOL [46]. The structural stability was evaluated through RMSD, radius–of–gyration (*R*_g_), and root–mean–square fluctuations (RMSF) computed using the “gmx rms”, “gmx rmsf”, and “gmx gyrate” tools of GROMACS, respectively. The intramolecular hydrogen bonds and secondary structure of MERTK were calculated using “gmx hbond” and “gmx do_dssp” [58] utilities of GROMACS. Further, the clustering was achieved using the Daura et al. algorithm with “gmx cluster” tool at an RMSD cut-off of 0.18 nm [59]. The principal component analysis (PCA) was used to evaluate the dynamic concerted motions of MERTK alone and MERTK complexes with GROMACS tools as described in our previous study. The free energy landscapes (FELs) [60,61] were generated utilizing the Boltzmann relation with “gmx sham” tool:


$$G_{\left(\text{PC1},\text{ PC2}\right)}\;=-k_{\text{B}}T\text{ ln }P_{\left(\text{PC1},\text{ PC2}\right)}$$ (1)

Where G represents Gibbs free energy, k_B_ signifies the Boltzmann constant, T denotes the absolute temperature, and P stands for the probability distribution of systems along PC1 and PC2. At last, the binding free energy was estimated employing the MM-PBSA approach with g_mmpbsa [62]. During the binding free energy estimation, the assessment of conformational entropy was not considered as per the previous literature studies [63,64].

## 3. Results and discussion

### 3.1. Molecular docking

The biological processes are depending on the interactions between ligand and protein with binding and detachment being critical activities. Higher binding affinity is linked to longer intervals between these instances. The investigation of protein ligand interactions sheds light on atomistic interactions, such as ligand binding and detachment from active sites.

**Fig 1:**
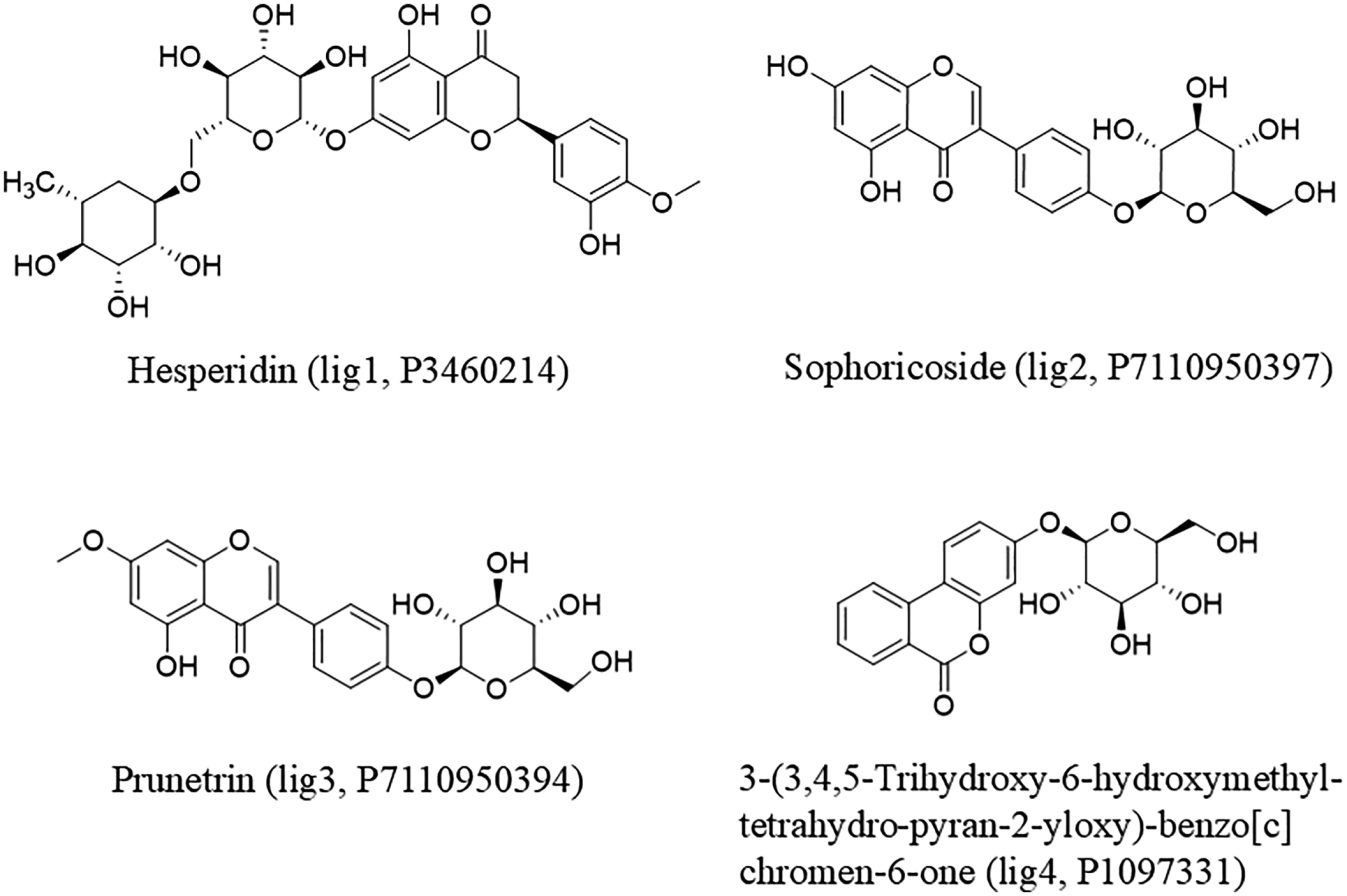
The 2D chemical structures of top hit ligands.

The present investigation used molecular docking to recognize how MERTK protein interacts with known and unknown inhibitors at structural level. UNC2025 reported compound was used as a positive control to see their interaction with targeted protein [37], and 1million compounds were retrieved from different databases such as ZINC, ChEMBL, and OTAVA chemical compound databases [34–36]. The MERTK protein was docked with the reported compound and new compounds through MOE 2015 for initial screening [44], and redocked the highest energy compounds to select top 10 compounds for further analysis. Molecular docking optimizes internal molecular contacts in protein ligand complexes, modulating binding configuration and inhibitory activities. After initial docking top 10 compounds were redocked with active site of MERTK protein, and four top inhibitors were selected on the basis of their high binding energy and hydrogen, van der Waals, and hydrophobic interactions. The docking method determined the best docked poses and interactions of compounds with various amino acids, which are essential for binding. Identifying ligands with the lowest binding affinity is typically critical in drug designing. In this investigation, we focused on one reported compound and the top four screened compounds selected from a library of one million compounds. The MERTK interacted with lig1 (P3460214), lig2 (P7110950397), lig3 (P7110950394), and lig4 (P1097331), exhibiting the highest binding energy (Fig 1). It was discovered that the MERTK has a standard protein kinase fold consisting of N and C lobes got involved by a hinge region and gatekeeper residue [10]. The ATP-binding pocket, which is crucial for interactions, has been identified between the N and C lobes, surrounded by the hinge region and G-loop [11]. The specified protein had a total structural mass of 35.07 kDa. These discoveries were the context for an inclusive recognition of the structural properties of MERTK and its potential interactions with ligands, as investigated by computational techniques [65].

**Fig 2:**
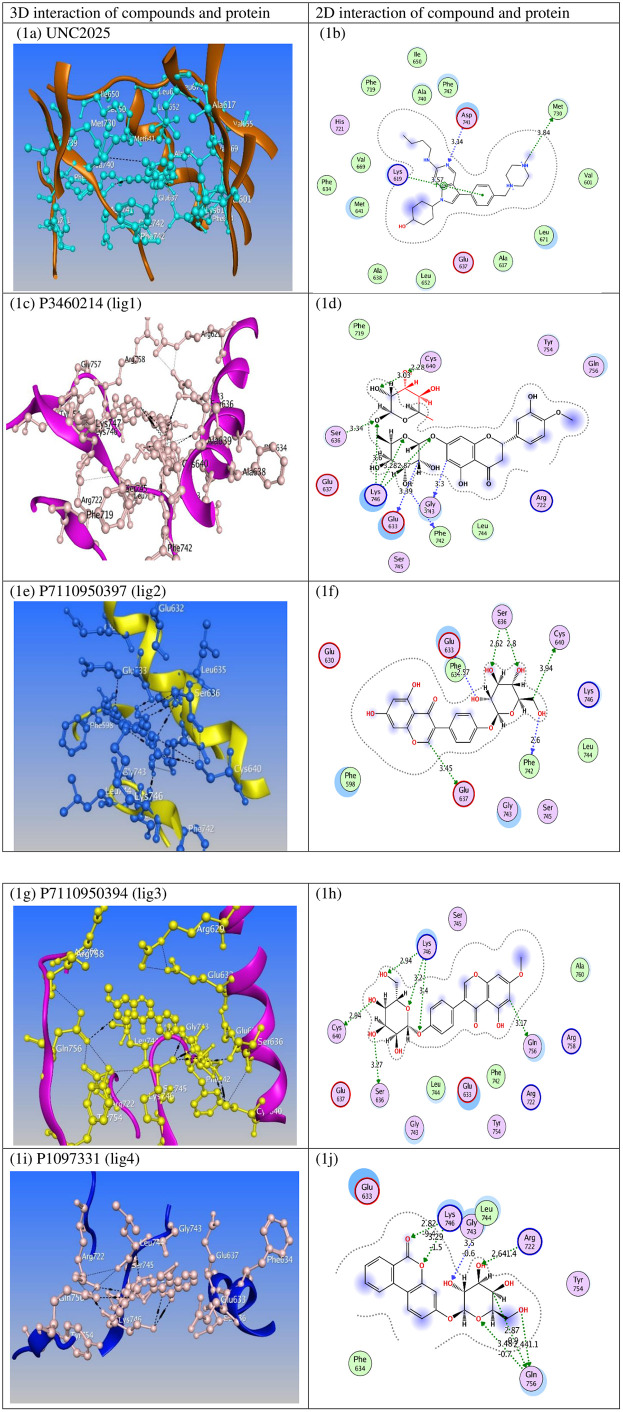
The hydrogen bond and hydrophobic interactions of MERTK complexes.

The UNC2025 compound (used as a positive control) interacted with MERTK for which binding affinity was obtained –12.436 kcal/mol (Table 1). In this investigation, we observed the hydrogen bonds of UNC2025 with Met730, Asp741 residues of MERTK (Fig 2a, Table 1). The residues Met641, Leu652, Leu671, Phe719, Ala740, Phe742 (blue shadow) showed van der Waals interactions, some residues (Val601, Ala617, Phe634, Glu637, Ala638, Ile650, Val669, His721) showed hydrophobic interactions, and Lys619 displayed cation-π interactions with UNC2025 (Fig 2b). After the docking, the docked complex of MERTK–UNC2025 was submitted for simulation to understand their stability with protein.

**Table 1:** Compounds with their binding energy exhibiting bonded interactions, van der Waals, and hydrophobic interactions.

Compound	Energy	bonded interactions	Distance	Angles	van der Waals	Hydrophobic interactions
UNC2025	–12.436	Lys619	3.57 Å	150.0˚	Met641, Leu652, Leu671, Phe719, Ala740, Phe742	Val601, Ala617, Phe634, Glu637, Ala638, Ile650, Val669, His721
		Met730	3.84 Å	97.3˚		
		Asp741	3.14 Å	130.7˚		
P3460214 (lig1)	–22.977	Glu633	3.39 Å	143.7˚	Leu744, Tyr754, Gln756	Glu637, Phe719, Arg722, Ser745
		Ser636	3.34 Å	74.2˚		
		Cys640	2.28 Å	64.7˚		
			3.03 Å	46.8˚		
		Phe742	3.00 Å	138.8˚		
		Gly743	3.30 Å	64.8˚		
		Lys746	2.87 Å	68.3˚		
			3.28 Å	70.3˚		
			3.60 Å	90.2˚		
P7110950397 (lig2)	–21.534	Glu633	2.57 Å	130.0˚	Phe598, Phe634, Gly743, Lys746	Glu630, Leu744, Ser745
		Ser636	2.62 Å	47.1˚		
			2.80 Å	68.2˚		
		Glu637	3.45 Å	97.9˚		
		Cys640	3.94 Å	84.8˚		
		Phe742	2.60 Å	158.9˚		
P7110950394 (lig3)	–19.872	Ser636	3.27 Å	152.8˚	Glu633, Gly743, Leu744, Ala760	Glu637, Arg722, Phe742, Ser745, Tyr754, Arg758
		Cys640	2.94 Å	132.6˚		
		Lys746	2.94 Å	113.8˚		
			3.20 Å	139.1˚		
			3.40 Å	173.4˚		
		Gln756	3.17 Å	113.0˚		
P1097331 (lig4)	–18.707	Arg722	2.64 Å	76.5˚	Glu633, Leu744	Phe634, Tyr754
		Gly743	3.50 Å	93.2˚		
		Lys746	2.82 Å	166.6˚		
			3.29 Å	138.7˚		
		Gln756	2.44 Å	135.3˚		
			2.87 Å	58.1˚		
			3.48 Å	149.3˚		

The lig1 was noted with highest binding affinity with MERTK out of top 10 hits, which showed the docking score of –22.977 kcal/mol (Table 1). Hydrogen bonds were observed between lig1 and Glu633, Ser636, Cys640, Phe742, Gly743, Lys746 residues of the protein (Table 1), while Leu744, Tyr754, Gln756 showed van der Waals interaction, and Glu637, Phe719, Arg722, Ser745 displayed hydrophobic interactions with lig1 as shown in Fig 2 (c,d). The second highest compound is lig2, which showed binding energy of –21.534 kcal/mol and formed hydrogen bonds with Glu633, Ser636, Phe742 residues of MERTK (Fig 2e, Table 1). Whereas, Phe598, Phe634, Gly743, Lys746 showed van der Waals interaction, and Glu630, Leu744, Ser745 formed hydrophobic contacts with lig2 as displayed in Fig 2f. The lig3 complex with MERTK has a binding energy of –19.872 kcal/mol (Table 1), which is found to be lower than that of lig1 and lig2.

However, lig3 exhibited strong interactions with MERTK protein and formed many hydrogen bonds with Ser636, Cys640, and Lys746 residues of MERTK as shown in Table 1 and Fig 2g. The van der Waals interaction was observed with Glu633, Gly743, Leu744, Ala760, and hydrophobic contacts were also observed between lig3 and Glu637, Arg722, Phe742, Ser745, Tyr754, Arg758 residues of MERTK (Fig 2h). The docking score of the lig4 compound is noted to be –18.707 kcal/mol, which formed hydrogen bond interactions with Arg722, Gly743, Lys746, Gln756 residues of MERTK (Fig 2i, Table 1). we also observed van der Waals interaction in Glu633 and Leu744 residues. The residues Phe634 and Tyr754 of MERTK were found to interact with lig4 through the hydrophobic contacts (Fig 2j). The observed key interactions between the top hit compounds and MERTK aligns well the study by Lambo et al., where various binding site residues were participating in hydrogen bond interaction and hydrophobic contacts with natural and conjugated inhibitors [32]. All the unknown compounds exhibited highest binding affinity, hydrogen bonding, hydrophobic contacts, and van der Waals interaction as compared to UNC2025, which were further subjected to MD simulations.

### 3.2. Structural stability and solvent accessibility for MERTK

**Fig 3:**
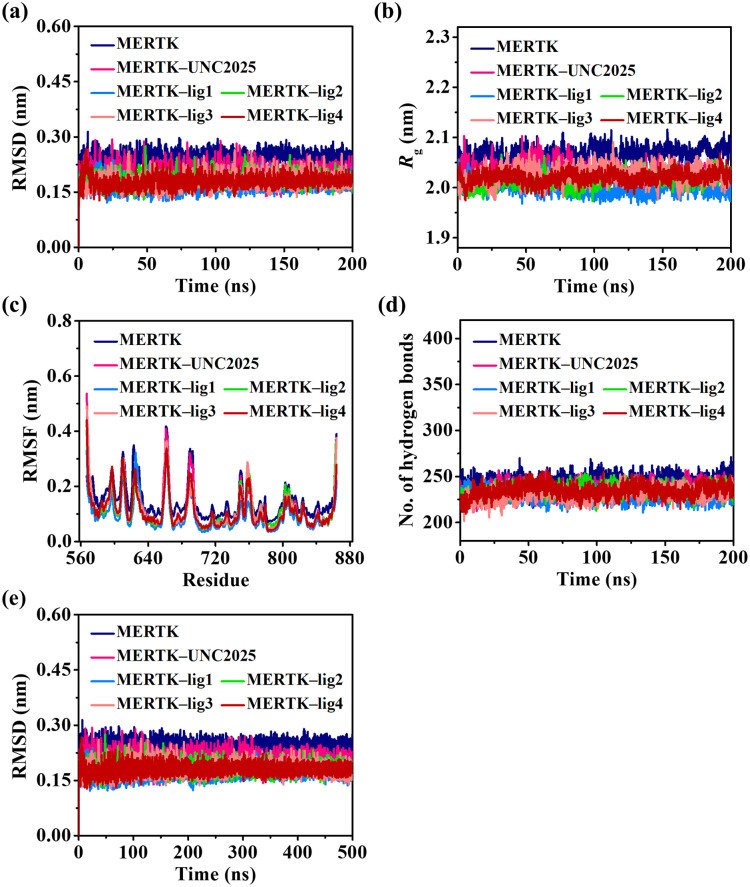
Global stability indices for MERTK protein and its complexes assessed through molecular dynamics simulations. (a) RMSD of all backbone atoms; (b) Rg of protein; (c) RMSF of all Cα atoms; and (d) Averaged number of intramolecular hydrogen bonds formed by amino acids, of MERTK for all systems during 200 ns MD simulations. (e) RMSD plot for MERTK and MERTK complexes for the 500 ns MD simulation.

To obtain atomistic insights, the MERTK complexes were submitted to 200 ns MD simulations using GROMACS for scrutinization of the structural conformation changes and atomic movements. The stability analyses of MERTK were done using RMSD, *R*_g_, and RMSF in the existence and non-existence of top-hit compounds including the reference compound UNC2025. In MERTK alone, the RMSD alters at a higher average RMSD of 0.246 nm (Fig 3a, Table 2). Whereas, the RMSD profile of MERTK complexes becomes stable on incorporation of UNC2025, lig1, lig2, lig3, and lig4, despite MERTK alone (Fig 3a). The average RMSD of MERTK–UNC2025, MERTK–lig1, MERTK–lig2, MERTK–lig3, and MERTK–lig4 was noted to be 0.201, 0.170, 0.183, 0.178, and 0.179 nm, respectively (Table 2). From the average RMSD and its plateau, it has been observed that lig1 extremely stabilized the structure of MERTK as compared to other compounds. Further, MD simulations of each system are extended to 500 ns to confirm the stability of complexes. Then, a subset of systems extended to 500 ns MD simulations was compared with the original 200 ns trajectories. The RMSD plot indicates that all the ligand-bound MERTK complexes (MERTK–UNC2025, MERTK–lig1, MERTK–lig2, MERTK–lig3, and MERTK–lig4) reached equilibrium and maintained stable RMSD average values with similar profiles during 200 ns as well as 500 ns simulations (Fig 3e), with notably reduced fluctuations compared to MERTK alone.

**Table 2:** The average RMSD, *R*_g_, RMSF, intramolecular hydrogen bonds, and trace values of PCA for all MERTK systems are listed.

Model system	Average			H-bond	PCA (nm2)
	RMSD (nm)	*R*_g_ (nm)	RMSF (nm)		
MERTK	0.246	2.068	0.149	241.863	5.705
MERTK–UNC2025	0.201	2.031	0.117	235.926	5.480
MERTK–lig1	0.170	2.000	0.094	229.060	3.814
MERTK–lig2	0.183	2.014	0.113	234.881	5.014
MERTK–lig3	0.178	2.025	0.116	231.176	5.444
MERTK–lig4	0.179	2.020	0.111	234.917	5.430

The plot of *R*_g_ illustrates the compactness of MERTK in alone and when complexed with UNC2025, lig1, lig2, lig3, and lig4 compounds. As displayed in Fig 3b, MERTK alone varies at a higher *R*_g_ average of 2.068 nm. Though upon incorporating UNC2025, lig1, lig2, lig3, and lig4 compounds, the *R*_g_ average was reduced to 2.031, 2.000, 2.014, 2.025, and 2.020 nm, respectively (Table 2). The reduced *R*_g_ average and its plateau displays the high compactness of MERTK in the existence of compounds as compared to MERTK alone (Fig 3b). Overall, the MERTK–lig1 complex exhibited less *R*_g_ fluctuation, indicating the higher compactness of MERTK on adding lig1.

RMSF was computed to inspect residual flexibility throughout the simulated duration, tracking the variability of each amino acid as they interact with the ligand along the trajectory. The residue-based RMSF plots for MERTK alone and MERTK complexes are showcased in Fig 3c. The averaged RMSF for MERTK alone was observed to be 0.149 nm and for MERTK–UNC2025, MERTK–lig1, MERTK–lig2, MERTK–lig3, MERTK–lig4 was calculated to be 0.117, 0.094, 0.113, 0.116, and 0.111 nm, respectively (Table 2). The residue-based RMSF analysis illuminated reduced fluctuations in MERTK residues, when UNC2025, lig1, lig2, lig3, and lig4 were added, compared to MERTK alone (Fig 3c). Intriguingly, the binding of lig1 to MERTK significantly decreased the fluctuations in the MERTK residues in comparison to the binding of other ligands.

### 3.3. Intramolecular hydrogen bond estimation in MERTK and MERTK complexes

The intramolecular hydrogen bonds are of paramount importance for stabilizing and maintaining the native structure of a protein [66,67]. The intramolecular hydrogen bonds calculated for MERTK alone and on the inclusion of screened hits along with reference compound UNC2025 are displayed in Fig 3d. In MERTK, the intramolecular hydrogen bonds were calculated to be 241.863, which decreased on the inclusion of UNC2025, lig1, lig2, lig3, and lig4 to 235.926, 229.060, 234.881, 231.176, and 234.917, respectively. Remarkably, the average intramolecular hydrogen bonds noted for MERTK in the MERTK–lig1 complex indicated the higher stability of MERTK in the existence of lig1, compared to other complexes.

### 3.4. Conformational clustering and secondary structure analysis for MERTK and MERTK complexes

**Fig 4:**
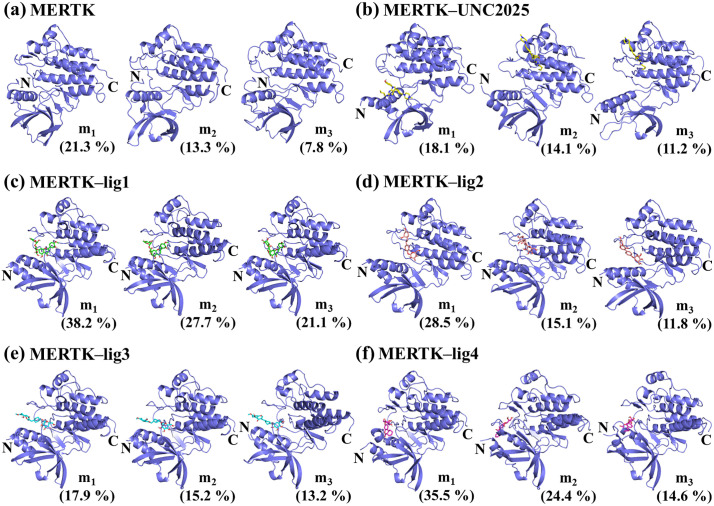
The top three representative members of conformational clusters for MERTK in both the free and bound states are highlighted as m_1_, m_2_, and m_3_, along with their percentage population.

The structural variation based clustering was conducted employing the Daura et al. algorithm [31] to depict the thermodynamic stability of MERTK alone and in the presence of top-hits. For MERTK alone, the percentage populations of the three most-populated microstates (m_1_, m_2_, m_3_) have been observed at 21.3%, 13.3%, and 7.8% (Fig 4a), however, they decreased to 18.1%, 14.1%, and 11.2%, respectively, for MERTK on incorporating UNC2025 (Fig 4b). As compared to MERTK alone and MERTK–UNC2025 complex, MERTK–lig1 exhibited 38.2%, 27.7%, and 21.1%, population in the three most populated microstates, respectively (Fig 4c).

Additionally, the percentage populations of the top three most-populated microstates were found to be 28.5%, 15.1%, 11.8%, respectively, in MERTK–lig2 complex; 17.9%, 15.2%, 13.2%, respectively, in MERTK–lig3; and 35.5%, 24.4%, 14.6%, respectively, in MERTK–lig4. [Fig 4 (d–f)]. The top three microstates accounted for 87%, 55.4%, 46.3%, and 74.5% of the whole conformational ensemble in MERTK–lig1, MERTK–lig2, MERTK–lig3, and MERTK–lig4 complexes, respectively, whereas only 42.4% and 43.4% of structures were sampled in the three most-populated microstates of MERTK alone and MERTK–UNC2025, respectively. Thus, the incorporation of lig1, lig2, lig3, and lig4 leads to the higher conformational homogeneity of the MERTK structure. The conformational clustering results indicated the greater thermodynamic stability of MERTK complexes in comparison of MERTK alone.

Furthermore, secondary structure analysis was conducted on the most populated clusters (m_1_, m_2_, m_3_). The representative members of the most-populated clusters of MERTK sampled helix (31%, 32%, 33%), β-sheet (22%, 19%, 19%), coil (21%, 23%, 22%), bend (11%, 12%, 12%), and turn (15%, 14%, 14%) as listed in Table 3. In contrast, the inclusion of UNC2025, lig1, lig2, lig3, and lig4 (Table 3) induced variations in the secondary structure of MERTK, characterized by an enhanced helix and coil contents and reduced β-sheet content. These alterations suggest enhanced structural stability of MERTK with the addition of the top hits.

**Table 3:** The secondary structure components evaluated for the representative member of conformational clusters for MERTK free and bound states are listed.

Protein/Protein-ligand complexes	Conformation	Secondary structure component (%)				
		Coil	β–sheeta	Bend	Turn	Helixb
MERTK	m_1_	21	22	11	15	31
	m_2_	23	19	12	14	32
	m_3_	22	19	12	14	33
MERTK–UNC2025	m_1_	22	19	9	13	37
	m_2_	24	17	9	14	36
	m_3_	22	18	10	13	37
MERTK–lig1	m_1_	22	18	8	13	39
	m_2_	22	19	9	13	37
	m_3_	22	19	8	13	38
MERTK–lig2	m_1_	22	19	9	13	37
	m_2_	22	20	7	15	36
	m_3_	23	19	7	15	36
MERTK–lig3	m_1_	22	18	10	14	36
	m_2_	22	19	8	13	38
	m_3_	24	19	8	12	37
MERTK–lig4	m_1_	21	20	11	14	34
	m_2_	20	20	11	14	35
	m_3_	22	20	9	16	33

aβ–sheet = β–bridge + β–strand; bHelix = 3_10_–helix + α–helix + π–helix.

### 3.5. PCA for MERTK in the absence and presence of top-hit compounds

**Fig 5:**
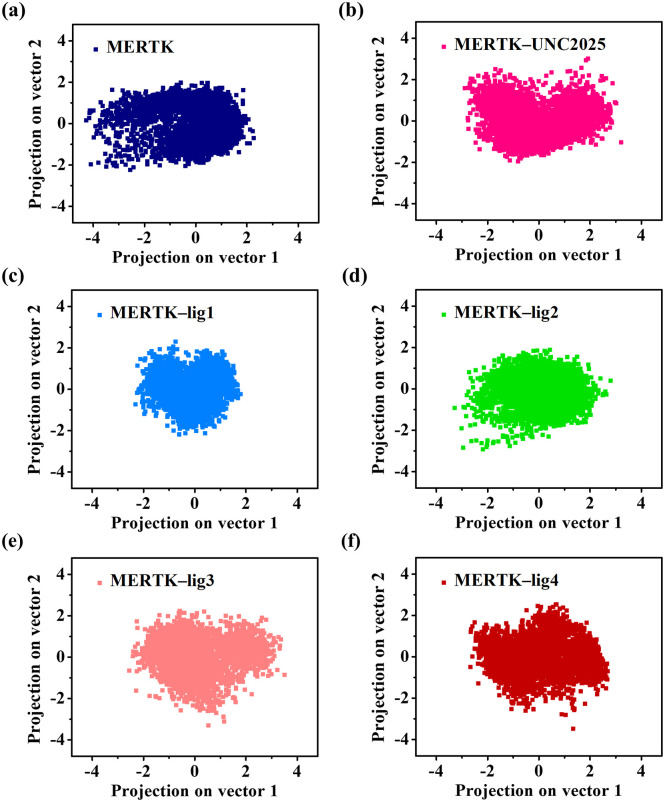
The PCA plots projected along vector 1 and vector 2 illustrate the dynamic motions of MERTK alone and complexes with UNC2025, lig1, lig2, lig3, and lig4 [panels (a–f)].

PCA is a covariance matrix method to examine the overall dynamic motions of a protein with and without top-hit compounds using the first two eigenvectors. The 2D projections of MD trajectories in the phase space province for MERTK and MERTK complexes are shown in Fig 5. The overall motions observed in the phase space region for MERTK–UNC2025, MERTK–lig1, MERTK–lig2, MERTK–lig3, and MERTK–lig4 complexes are lower than MERTK alone (Fig 5). Moreover, the conformational stability of all the systems was assessed by using trace values (Table 2). The estimated trace values for MERTK, MERTK–UNC2025, MERTK–lig1, MERTK–lig2, MERTK–lig3, and MERTK–lig4 were found to be 5.705 nm2, 5.480 nm2, 3.814 nm2, 5.014 nm2, 5.444 nm2, and 5.430 nm2, respectively. Notably, the trace values revealed the higher conformational stability of the MERTK–lig1 complex compared to both MERTK alone and other MERTK complexes.

### 3.6. FEL for MERTK in the absence and presence of top-hit compounds

**Fig 6:**
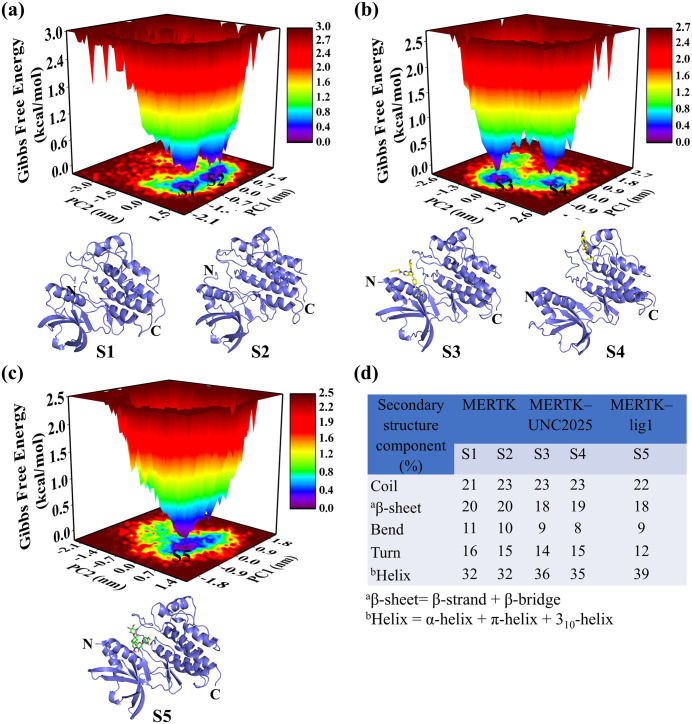
The FEL analyses of MERTK, MERTK–UNC2025, and MERTK–lig1 are displayed in panels (a–c), respectively. The conformations extracted correspond to minimum energy basins are shown as S1, S2, S3, S4, and S5. The secondary structure statistics of extracted conformations are displayed in panel **d**.

**Fig 7:**
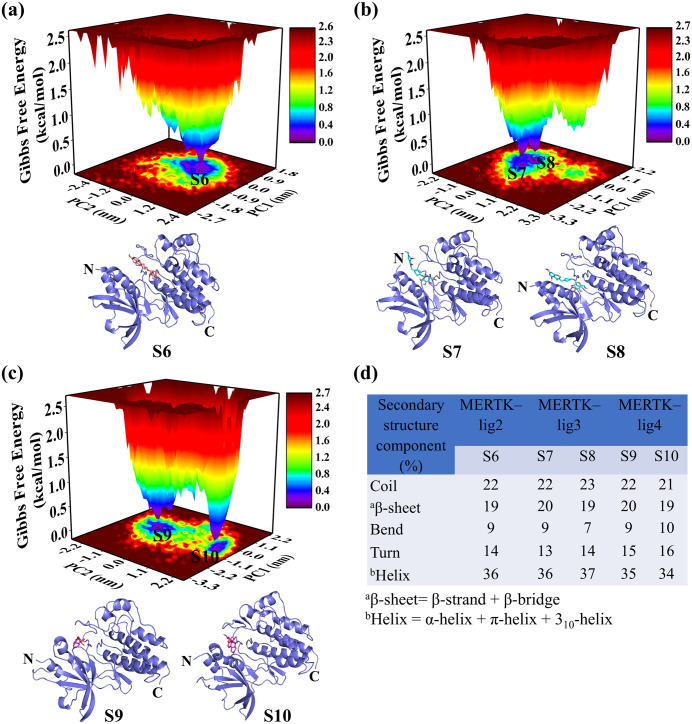
The FEL analyses of MERTK–lig2, MERTK–lig3, and MERTK–lig4 [panels (a–c)], respectively. The conformations extracted correspond to minimum energy basins are shown as S6, S7, S8, S9, and S10. The secondary structure statistics of extracted conformations are displayed in panel **d.**

To elucidate how top-hit compounds impact the conformational stability of MERTK, FELs were constructed using PC1 and PC2 as shown in Figs 6 and 7. FELs disclosed energies range between 0–3.0 kcal/mol for MERTK, 0–2.7 kcal/mol for MERTK–UNC2025, 0–2.5 kcal/mol for MERTK–lig1, 0–2.6 kcal/mol for MERTK–lig2, and 0–2.7 kcal/mol for MERTK–lig3 as well as MERTK–lig4. These results accentuate the diverse conformational subspaces sampled by each system, as illustrated in Figs 6 and 7.

The MERTK–lig1 and MERTK–lig2 exhibited a one global minimum with a higher number of minimum energy basins as compared to MERTK, MERTK–UNC2025, MERTK–lig3, and MERTK–lig4. The FEL results emphasized that lig1 and lig2 prominently modulated the conformational space of MERTK. The minimum-energy conformation S1 and S2 extracted from the FEL of MERTK alone sampled (20%, 20%) β-sheet, (21%, 23%) coil, (11%, 10%) bend, (16%, 15%) turn content, and (32%, 32%) helix, (Fig 6d).

However, the S3 and S4 conformations extracted from MERTK–UNC2025 sampled [18%, 19% β-sheet; 23%, 23% coil; 9%, 8% bend; 14%, 15% turn content; and 36%, 35% helix; (Fig 6d)], S5 conformation extracted from MERTK–lig1 sampled [18% β-sheet, 22% coil, 9% bend, 12% turn content, and 39% helix, (Fig 6d)], S6 conformation extracted from MERTK–lig2 [19% β-sheet, 22% coil, 9% bend, 14% turn content, and 36% helix (Fig 7d)], S7 and S8 conformation extracted from MERTK–lig3 [20%, 19% β-sheet, 22%, 23% coil, 9%, 7% bend, 13%, 14% turn content, and 36%, 37% helix (Fig 7d)], S9 and S10 conformation extracted from MERTK–lig4 [20%, 19% β-sheet, 22%, 21% coil, 9%, 10% bend, 15%, 16% turn content, and 35%, 34% helix (Fig 7d)]. Noticeably, the intense blue area in MERTK complexes portrayed the conformations with increased helix content, decreased β-sheet, and turn contents, highlighting the steadiness of MERTK structure in the presence of top-hits compared to MERTK alone.

### 3.7. Binding free energy calculations

The MM-PBSA method was utilized to calculate the binding free energy for evaluating the binding affinity and interactions of the top-hits with MERTK. Table 4 represents the computed components of the binding free energy. The findings of binding free energy calculations indicated that van der Waals interaction energy predominantly governs the binding free energy of the complexes.

**Table 4:** The binding free energy (ΔG_binding_) components (kcal/mol) between MERTK and top-hit compounds along with UNC2025 were estimated using MM-PBSA.

Energy components	MERTK–UNC2025	MERTK–lig1	MERTK–lig2	MERTK–lig3	MERTK–lig4
∆E_vdW_	–21.3 ± 2.3	–45.3 ± 4.0	–40.9 ± 3.1	–39.6 ± 2.7	–37.7 ± 3.6
∆E_elec_	–4.1 ± 1.9	–4.8 ± 1.9	–11.1 ± 2.4	–5.5 ± 1.9	–4.7 ± 2.8
∆E_MM_a	–25.4 ± 4.2	–50.1 ± 5.9	–52.0 ± 5.5	–45.1 ± 4.6	–42.4 ± 6.4
∆G_ps_	15.5 ± 10.8	27.9 ± 5.7	33.8 ± 5.3	20.1 ± 3.2	23.9 ± 5.8
∆G_nps_	–21.3 ± 9.7	–39.8 ± 5.3	–39.6 ± 6.6	–35.9 ± 4.6	–37.9 ± 6.1
∆G_solv_b	–5.8 ± 1.1	–11.9 ± 0.4	–5.8 ± 1.3	–15.8 ± 1.4	–14.0 ± 0.3
ΔG_binding_c	–31.2 ± 5.3	–62.0 ± 6.3	–57.8 ± 6.8	–60.9 ± 6.0	–56.4 ± 6.7

a∆E_MM_ = ∆E_vdW* *_+ ∆E_elec_; b∆G_solv_ = ∆G_ps* *_+ ∆G_nps_; c∆G_binding_ = ∆E_MM* *_+ ∆G_solv_.

**Fig 8:**
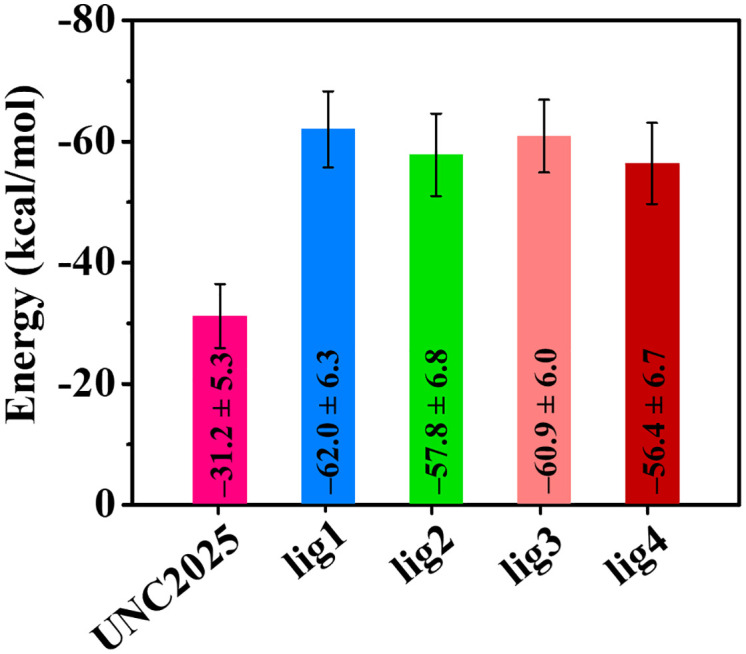
The estimated binding free energy between the MERTK residues and screened compounds, is derived from various interaction energy components.

Equally, electrostatic energy and non-polar solvation energies contributed favorably, while polar solvation energy exerts a detrimental impact on the binding free energy in the formation of MERTK complexes with the top three hits. As shown in Fig 8 and Table 4, lig1 reveals the highest binding free energy of –62.0 ± 6.3 kcal/mol, in its interaction with MERTK compared to lig2, lig3, and lig4 compounds. In conclusion, the MM-PBSA analysis of binding free energy underlines the substantial binding potential of lig1 with MERTK.

### 3.8. Residue-wise binding free decomposition energy

**Fig 9:**
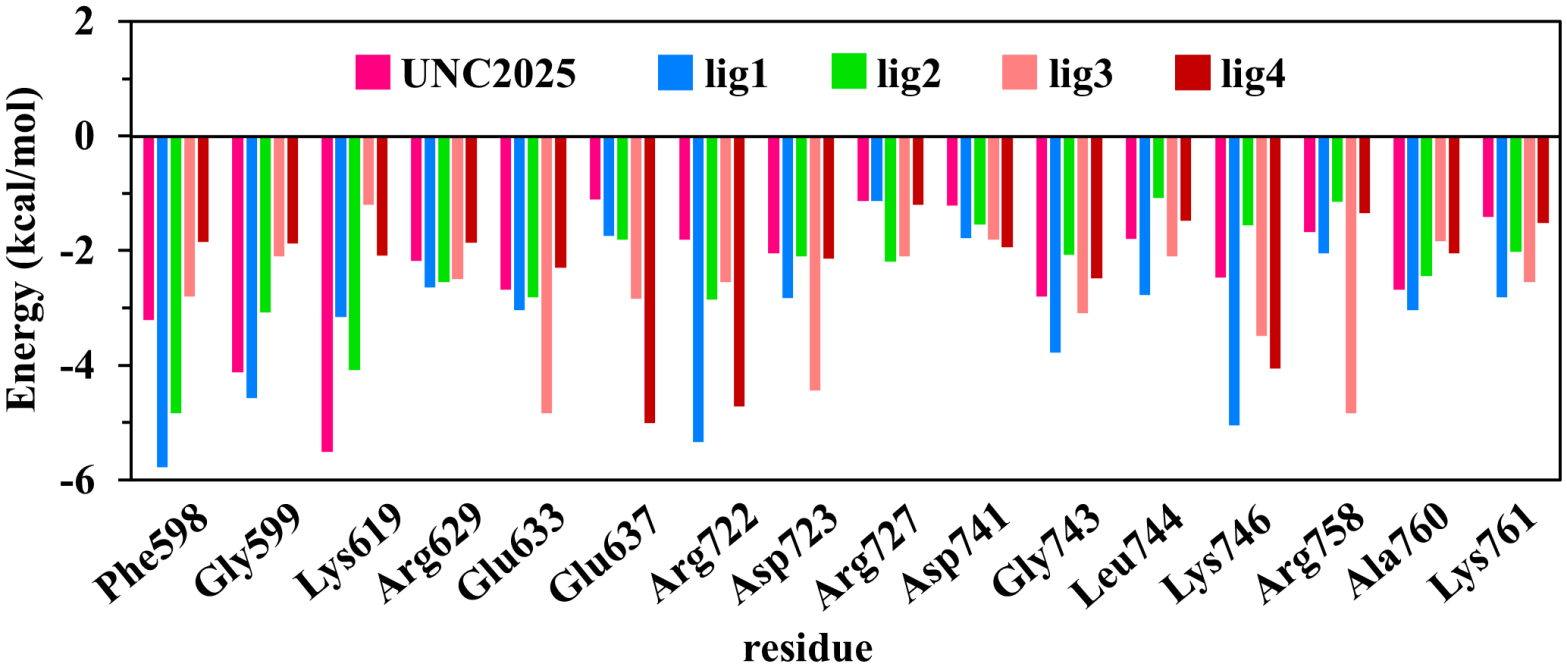
The residue-specific decomposition of binding free energy for MERTK with UNC2025, lig1, lig2, lig3 and lig4.

In addition to MM-PBSA, the calculated binding free energy was residue-wise decomposed for MERTK complexes as displayed in Fig 9. The investigation of residue-wise decomposed binding free energy identified the crucial residues that participated in the binding interaction of top-hits with MERTK. Fig 9 describes the substantial residues, including Phe598, Gly599, Lys619, Arg629, Glu633, Glu637, Arg722, Asp723, Arg727, Asp741, Gly743, Leu744, Lys746, Arg758, Ala760, and Lys761 of MERTK, which play a critical role in the complex formation of MERTK with UNC2025, lig1, lig2, lig3, and lig4. The residues contributing in the complex formation of MERTK with top hit candidates are in accordance with Zhang et al., where the interactions of various inhibitors were reported with G-loop (residues 594–598), hinge region (residues 672–679), catalytic loop residue 727, and A-loop (residues 744–762) of MERTK to form the complexes [31]. Altogether, residue-wise decomposed binding free energy analysis suggested the binding of top-hits with the specific residues within the binding site of MERTK, potentially representing active sites of MERTK for ligand binding.

In the present study, four compounds were shortlisted as potential hits, namely hesperidin (lig1, P3460214), sophoricoside (lig2, P7110950397), prunetrin (lig3, P7110950394), and c3-(3,4,5-trihydroxy-6-hydroxymethyl-tetrahydro-pyran-2-yloxy)-benzo[c]chromen-6-one (lig4, P1097331), based on their key binding interactions with MERTK. Hesperidin (lig1) is a citrus-derived flavanone glycoside comprising the aglycone hesperetin linked to a disaccharide rutinose. Its polyhydroxylated structure confers strong antioxidant capacity, and numerous studies have reported its anti-inflammatory, cardioprotective, neuroprotective, and anticancer properties [68–70]. These pharmacological benefits are largely mediated through free radical scavenging, suppression of pro-inflammatory cytokines, and regulation of cell-signaling pathways. Sophoricoside (lig2) is an isoflavone glycoside identified as genistein-4’-O-β-D-glucoside, predominantly present in *Styphnolobium japonicum* [71]. It has been documented to exhibit anti-inflammatory, anti-allergic, estrogenic, hepatoprotective, and anti-osteoporotic activities. Mechanistically, sophoricoside regulates immune responses by inhibiting mast-cell degranulation and attenuating Th2 cytokine production, highlighting its therapeutic potential.

Prunetrin (lig3), also known as prunetin-4’-O-β-D-glucoside, is a glycosylated isoflavone derived from *Prunus* species [72]. Beyond its antioxidant and anti-inflammatory roles, recent evidence demonstrates anticancer activity, particularly in hepatocellular carcinoma cells, where it induces G2/M phase arrest and apoptosis through activation of caspase cascades and inhibition of AKT/mTOR signaling. The lig4 is structurally characterized as 3-(3,4,5-trihydroxy-6-hydroxymethyl-tetrahydro-pyran-2-yloxy)-benzo[c]chromen-6-one [73]. Although its chemical structure has been elucidated, no substantial reports are available regarding its biological or pharmacological activities. Therefore, lig4 represents an unexplored scaffold that could offer novel opportunities for therapeutic investigation. Collectively, these hits represent a diverse set of glycosylated flavonoids and chromenone derivatives, several of which have documented bioactivities that align with antioxidant, anti-inflammatory, and anticancer pathways, while others remain underexplored, thereby offering scope for further evaluation.

Furthermore, based-on the key results of the present study, the possible future modifications in the structures of top hit ligands may be done as follows: In lig1 (Hesperidin), the disaccharide rutinose contributes to high polarity and poor permeability. Future analogues may involve mono-glycosylated or de-glycosylated derivatives (*e.g.*, hesperetin analogues), or selective *o*-methylation of hydroxyl groups to reduce polarity while retaining hydrogen-bonding potential. With a single glucose moiety, sophoricoside (lig2) is structurally simpler. Modifications could focus on alkylation or acylation of phenolic hydroxyl groups to improve lipophilicity and membrane permeability, while maintaining the isoflavone pharmacophore. The prunetin (lig3) core offers opportunities for substitution on the phenyl ring (*e.g.*, halogenation, methoxylation, or introduction of small hydrophobic groups) to enhance π–π stacking and hydrophobic contacts within the active site, potentially improving binding affinity. As an underexplored glycosylated chromenone derivative (lig4), structural simplification (*e.g.*, removal or modification of the sugar moiety) and ring substitutions on the chromenone core may be tested to improve lipophilicity and optimize active-site fit. Conclusively, the results indicate that glycosylated flavonoids and chromenone scaffolds form a promising chemical space for further optimization. The main strategies for improving predicted activity and drug-likeness include reducing excessive polarity through partial de-glycosylation or *o*-methylation, introducing small hydrophobic substituents to strengthen active-site interactions, and exploring simplified analogues of complex scaffolds such as hesperidin. These insights will serve as a rational basis for future lead optimization and drug development.

## 4. Conclusion

In this investigation, we used computational technique to find novel inhibitor for MERTK protein. We used molecular docking, MD simulations, ligand-based analysis, and free energy calculations using MM-PBSA, to identify probable drug targeting MERTK protein from different databases. The main goal of this research to identify therapeutic inhibitors with physiological significance, demonstrating the need for novel alternatives to therapy. We observed that the top hits showed higher binding affinity and stability for the MERTK protein for lig1 to lig4 as compared to neat inhibitor. The MERTK protein share the same active site for catalytic binding, and our study underscores the critical role of hydrogen bond, hydrophobic contacts and van der Waals interaction in the MERTK complexes. Molecular docking identified specific amino acids including Lys619, Ser636, Arg727, Asp741, Phe742, Lys746, and Gln756 as crucial residues for maintaining ligand stability with active site of protein. The RMSD and RMSF analyses demonstrated considerable conformational changes in the active region and its surroundings following ligand interaction. PCA and FEL sheds light on the reduced dynamic motions of MERTK proteins in the presence of top hit candidates. MMPBSA binding energy analysis highlights the robust binding of lig1 with MERTK (–60.0 kcal/mol) The PCA analysis result signifying the high stability in the binding of lig1 with MERTK protein. This one compound is most promising binder with MERTK protein with their significant binding affinity, individual interactions, and sustained presence within active pocket. Future validation through *in vitro* kinase assays, cell-based studies in MERTK-overexpressing cancer models, and *in vivo* xenograft evaluations will be crucial to confirm the therapeutic potential of the identified compounds. This research has potential to develop the multitarget drug focus of MERTK protein providing new therapeutic possibilities for the treating of variety of diseases.

## Supporting information

S1 File Figure TF formate. (ZIP)
