# Supplementary figures and images for: Targeting MERTK tyrosine kinase: Virtual screening and molecular dynamics insights for anti-cancer drug development
Source: PLoS One. 2025 Oct 30;20(10):e0334106. doi: 10.1371/journal.pone.0334106 (PMC12574917; doi:10.1371/journal.pone.0334106)

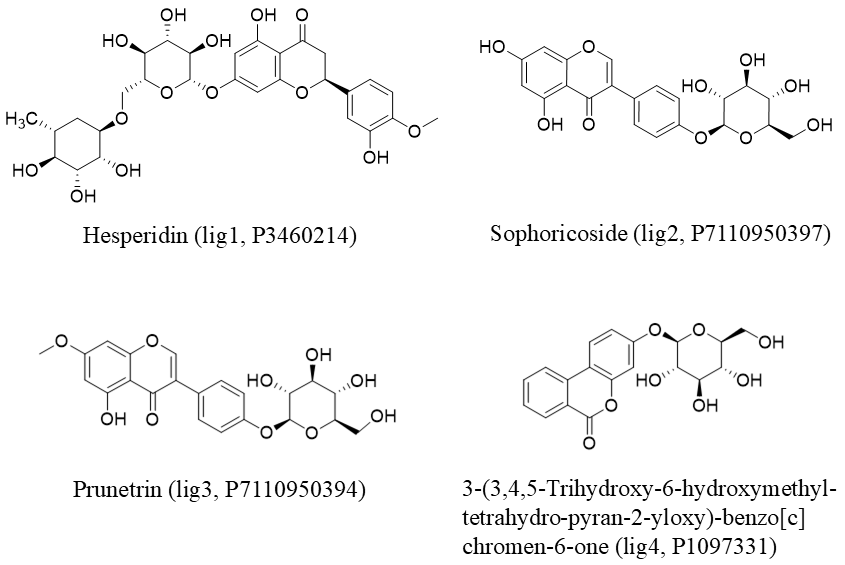

Supplement: S1 File — (ZIP) [file pone.0334106.s001.zip › figure tf formate/Fig 1.tif]

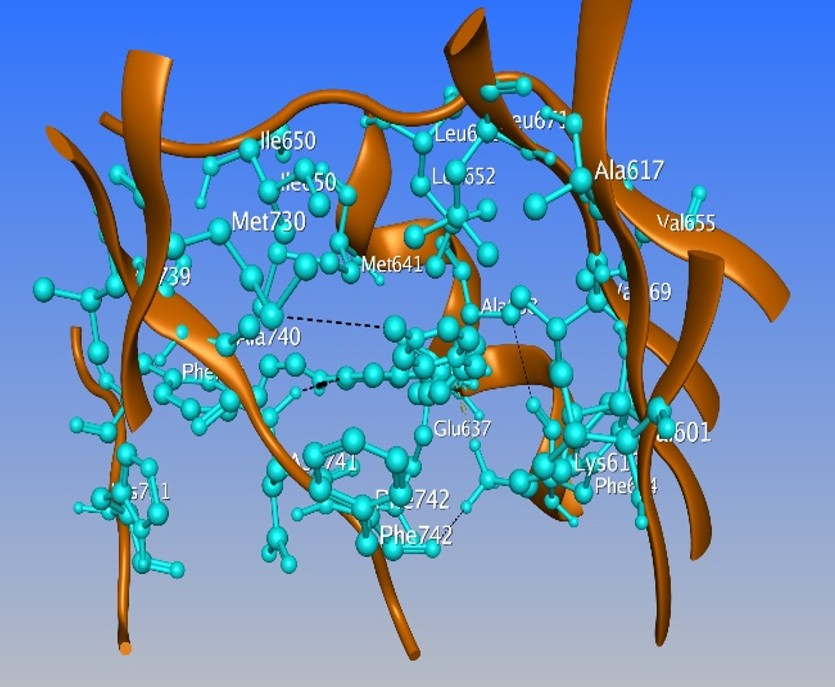

Supplement: S1 File — (ZIP) [file pone.0334106.s001.zip › figure tf formate/fig 1a.tiff]

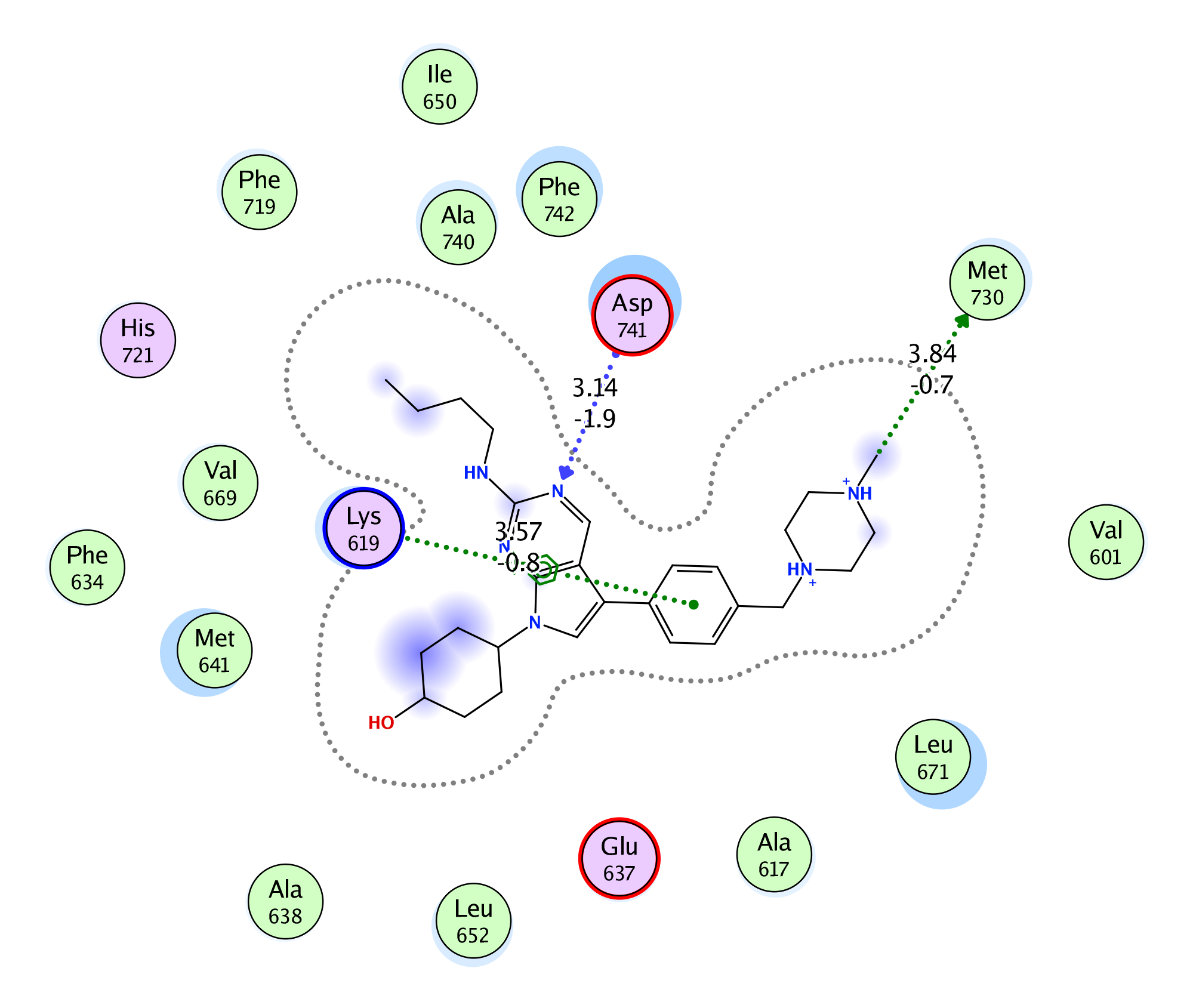

Supplement: S1 File — (ZIP) [file pone.0334106.s001.zip › figure tf formate/fig 1b.tiff]

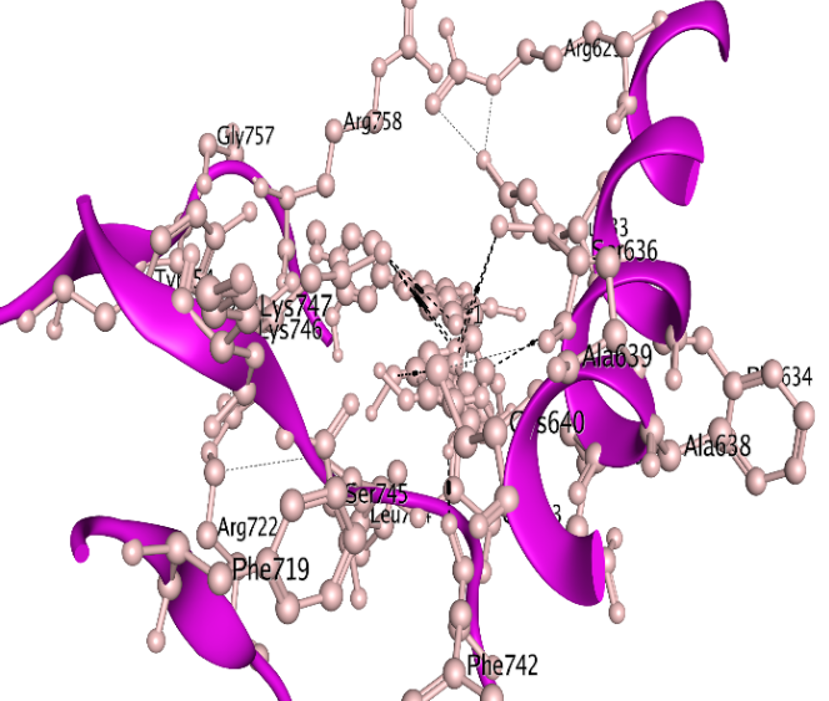

Supplement: S1 File — (ZIP) [file pone.0334106.s001.zip › figure tf formate/fig 1C.tif]

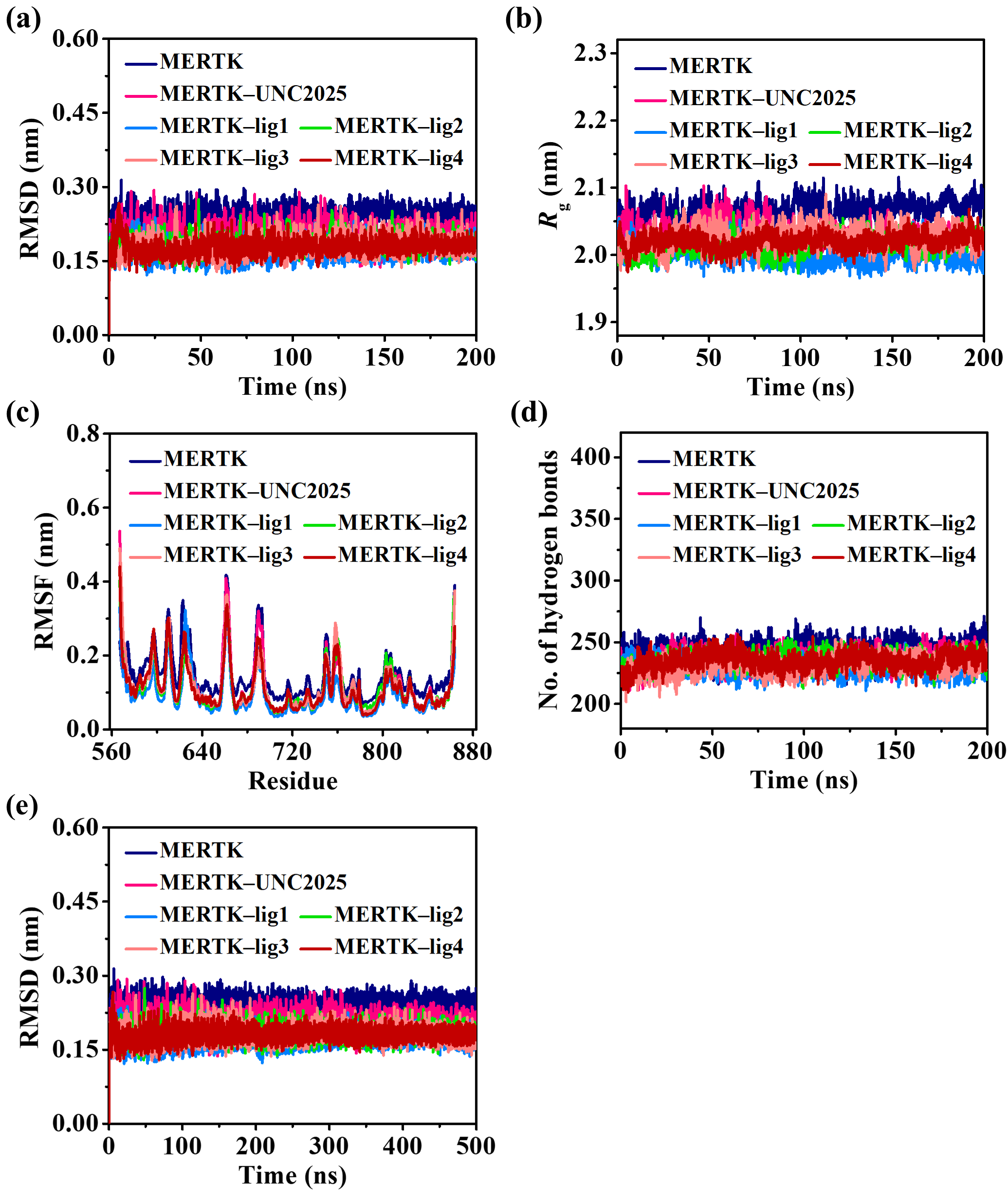

Supplement: S1 File — (ZIP) [file pone.0334106.s001.zip › figure tf formate/Fig 3.tif]

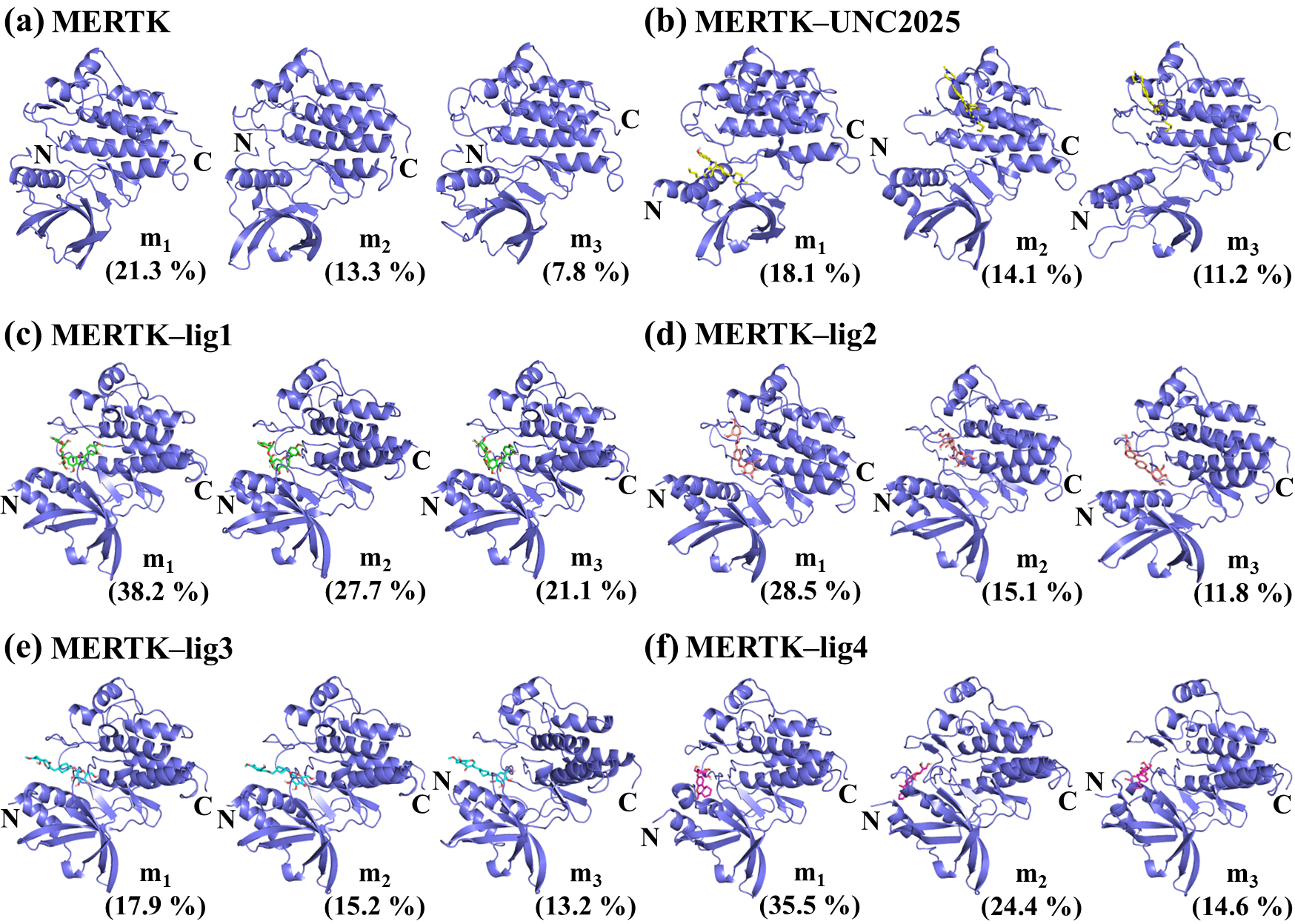

Supplement: S1 File — (ZIP) [file pone.0334106.s001.zip › figure tf formate/Fig 4.tif]

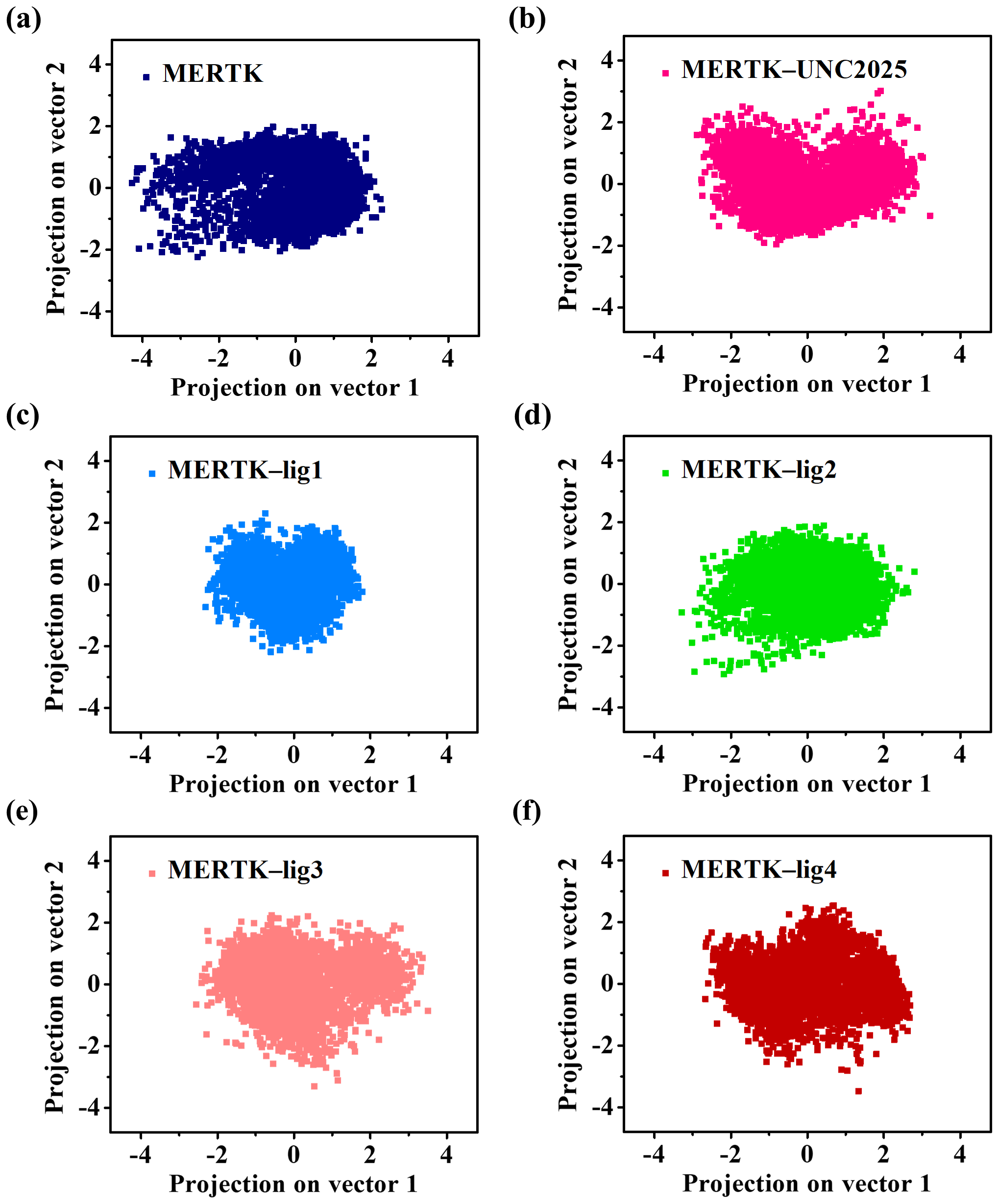

Supplement: S1 File — (ZIP) [file pone.0334106.s001.zip › figure tf formate/Fig 5.tif]

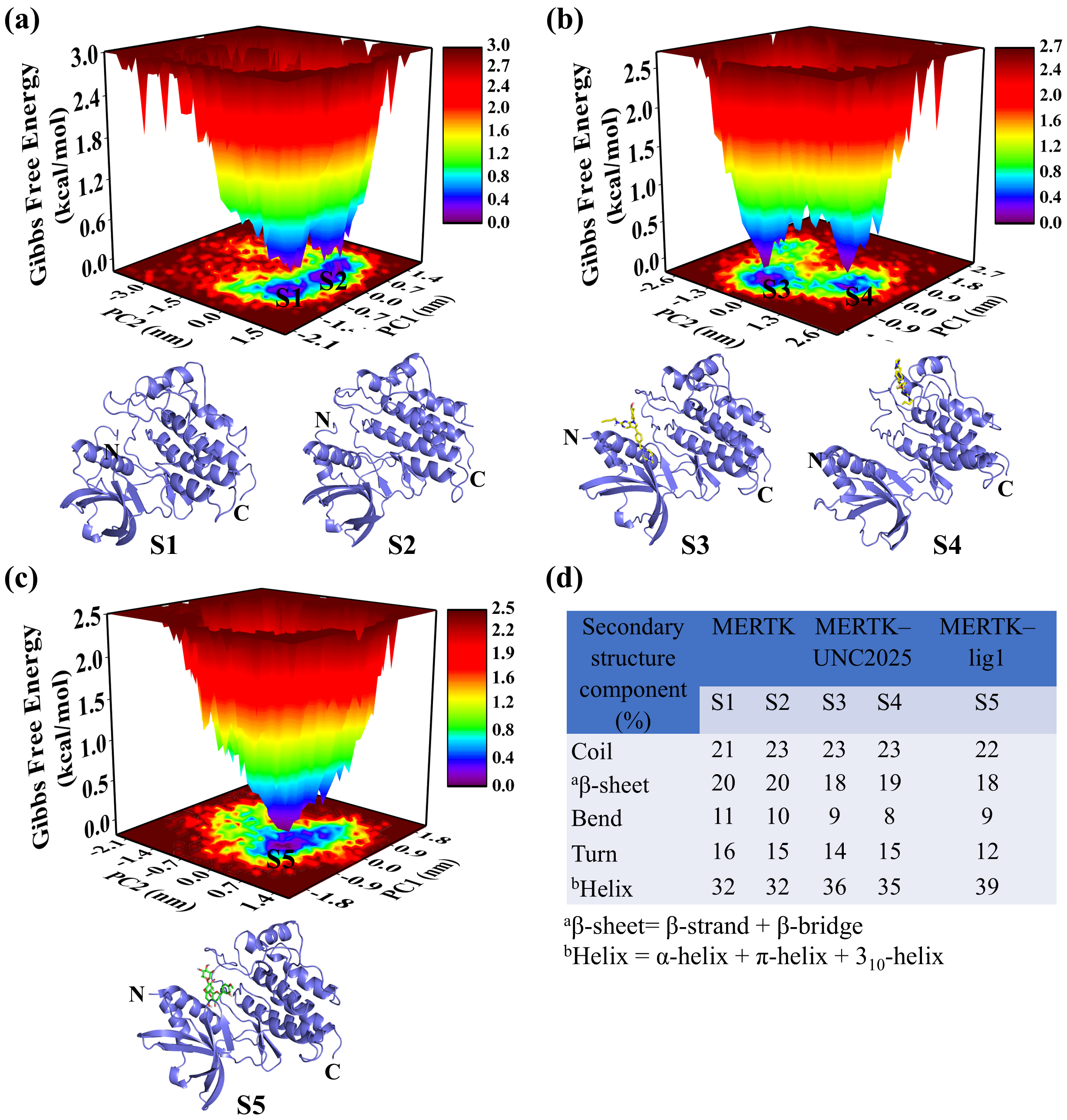

Supplement: S1 File — (ZIP) [file pone.0334106.s001.zip › figure tf formate/Fig 6.tif]

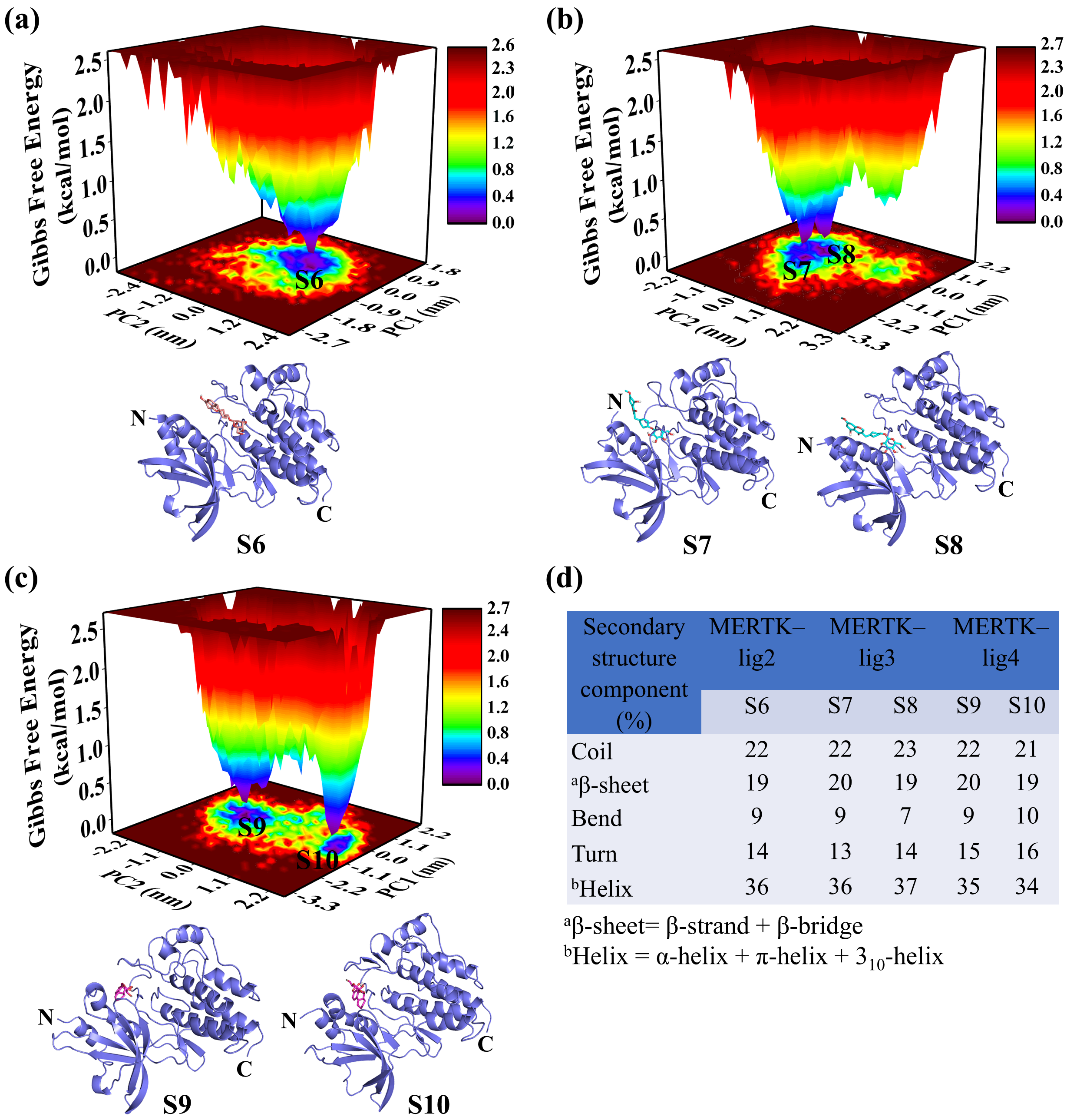

Supplement: S1 File — (ZIP) [file pone.0334106.s001.zip › figure tf formate/Fig 7.tif]

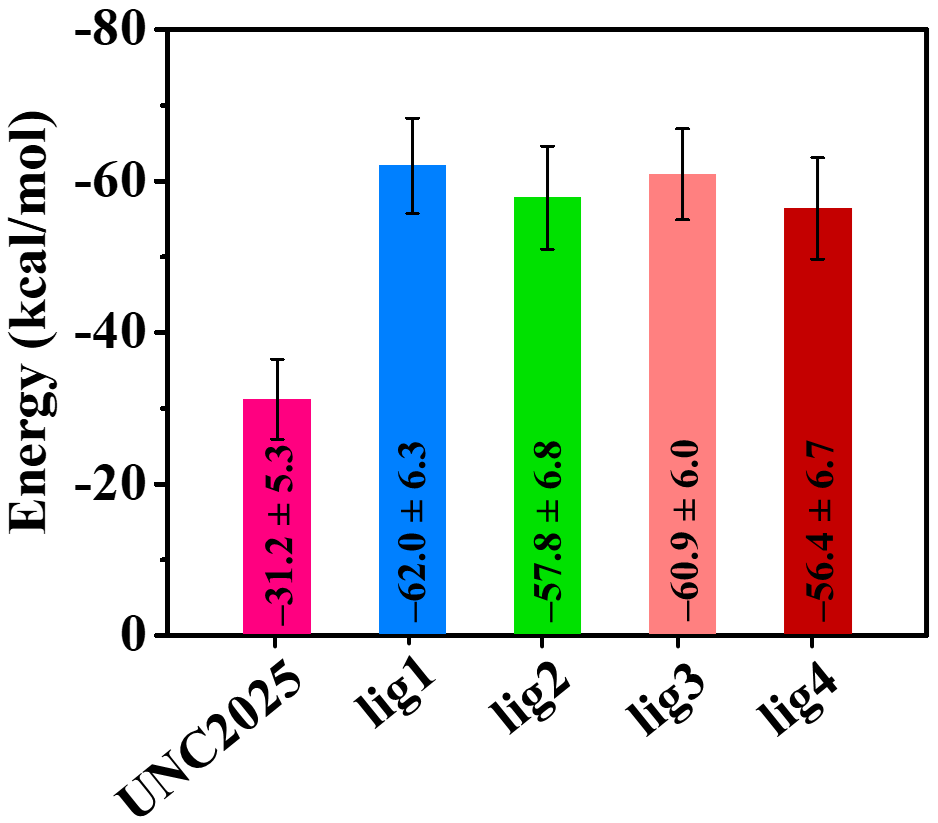

Supplement: S1 File — (ZIP) [file pone.0334106.s001.zip › figure tf formate/Fig 8.tif]

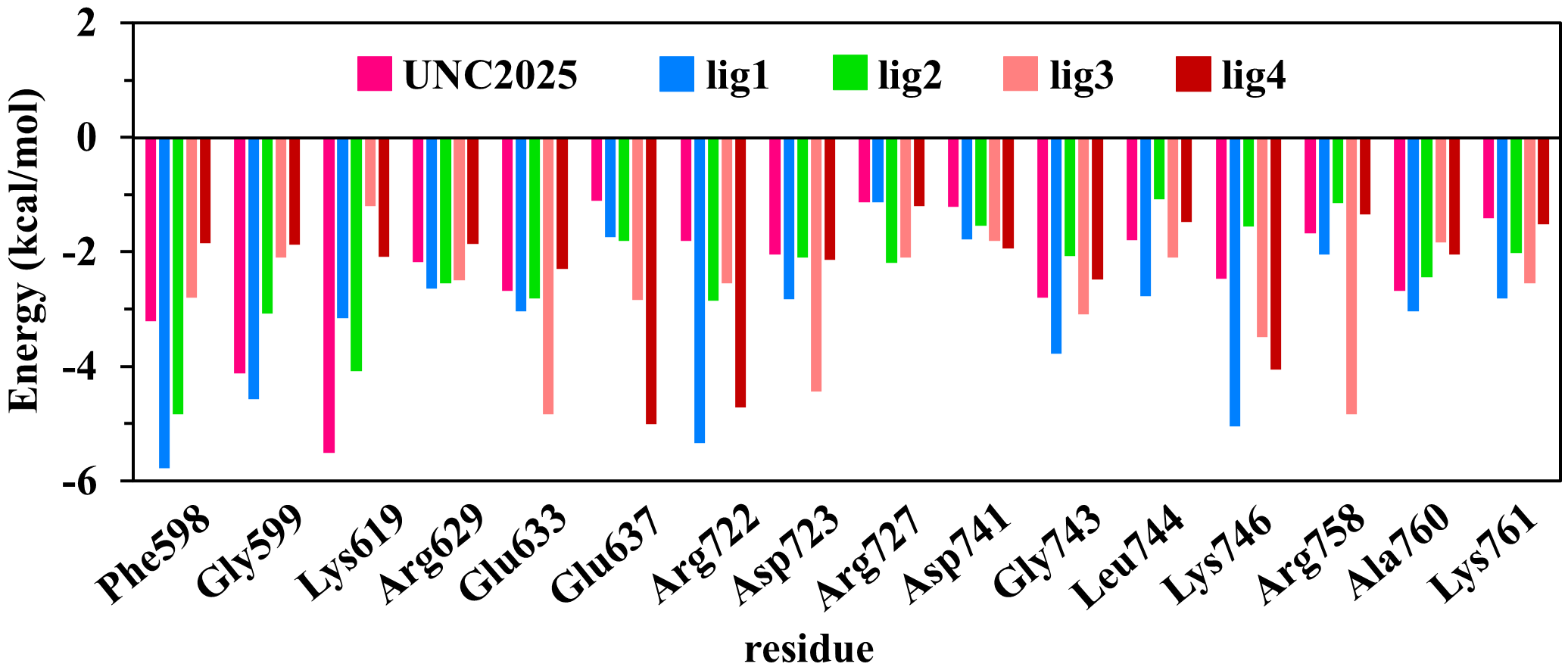

Supplement: S1 File — (ZIP) [file pone.0334106.s001.zip › figure tf formate/Fig 9.tif]

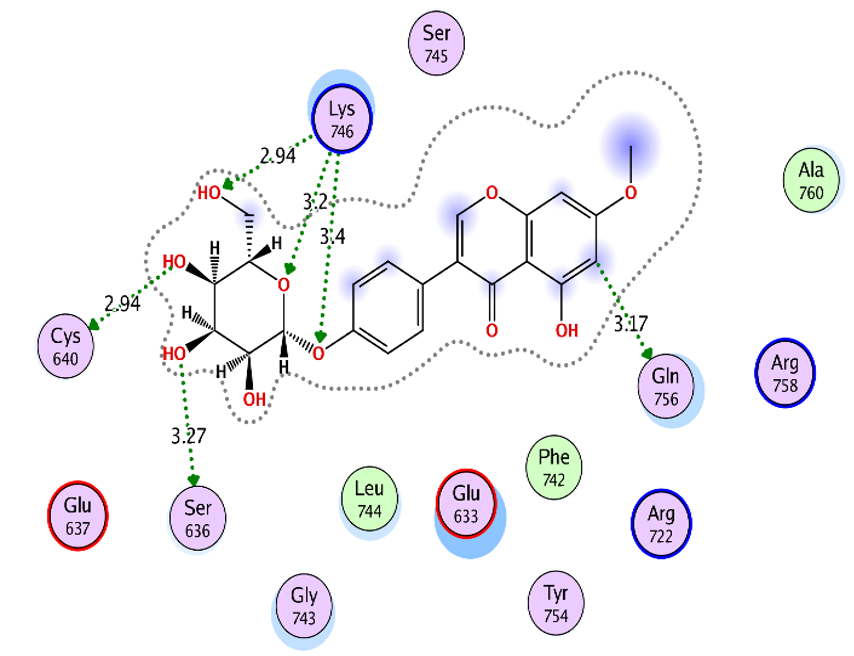

Supplement: S1 File — (ZIP) [file pone.0334106.s001.zip › figure tf formate/FIG,1H.tif]

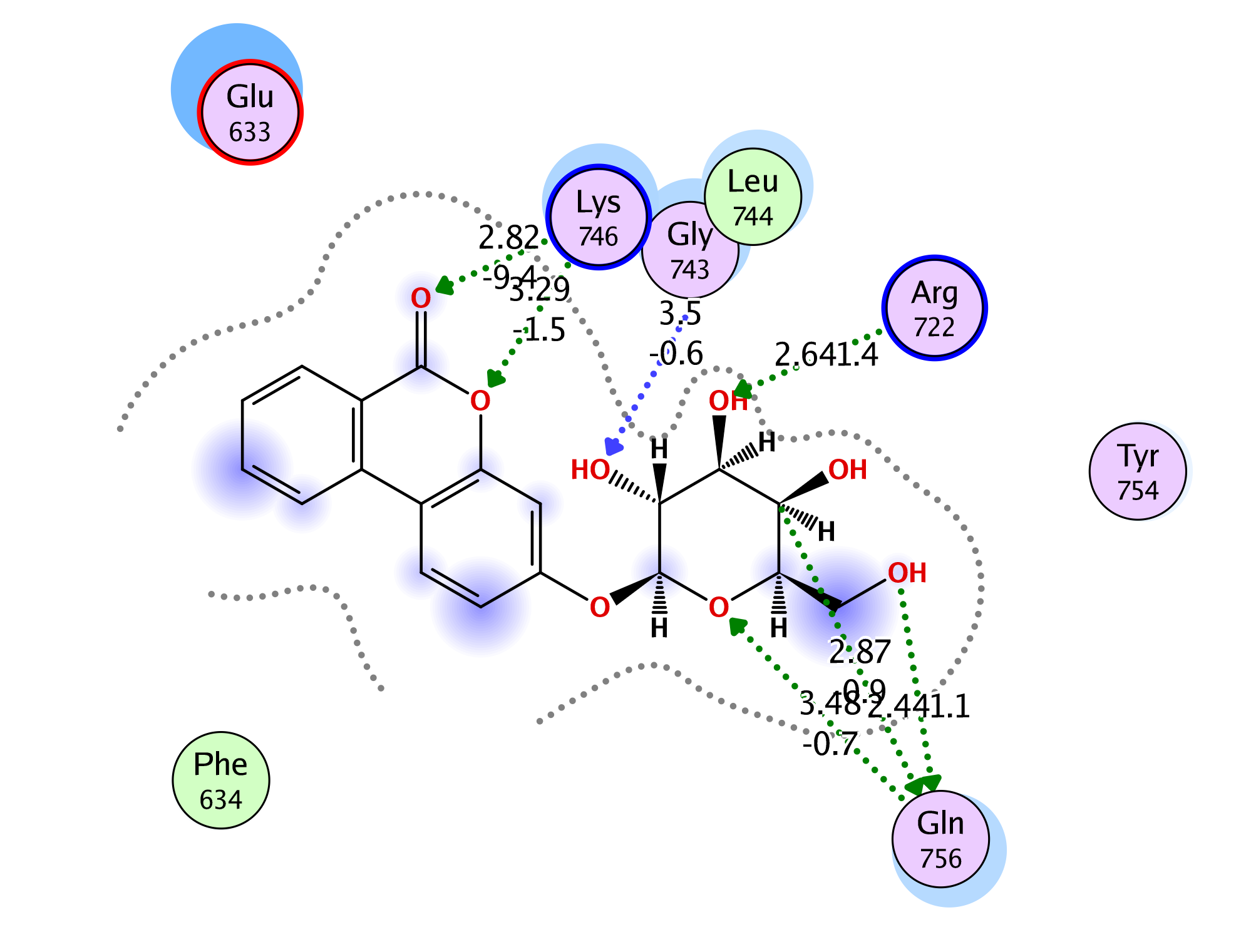

Supplement: S1 File — (ZIP) [file pone.0334106.s001.zip › figure tf formate/FIG1,J.tiff]

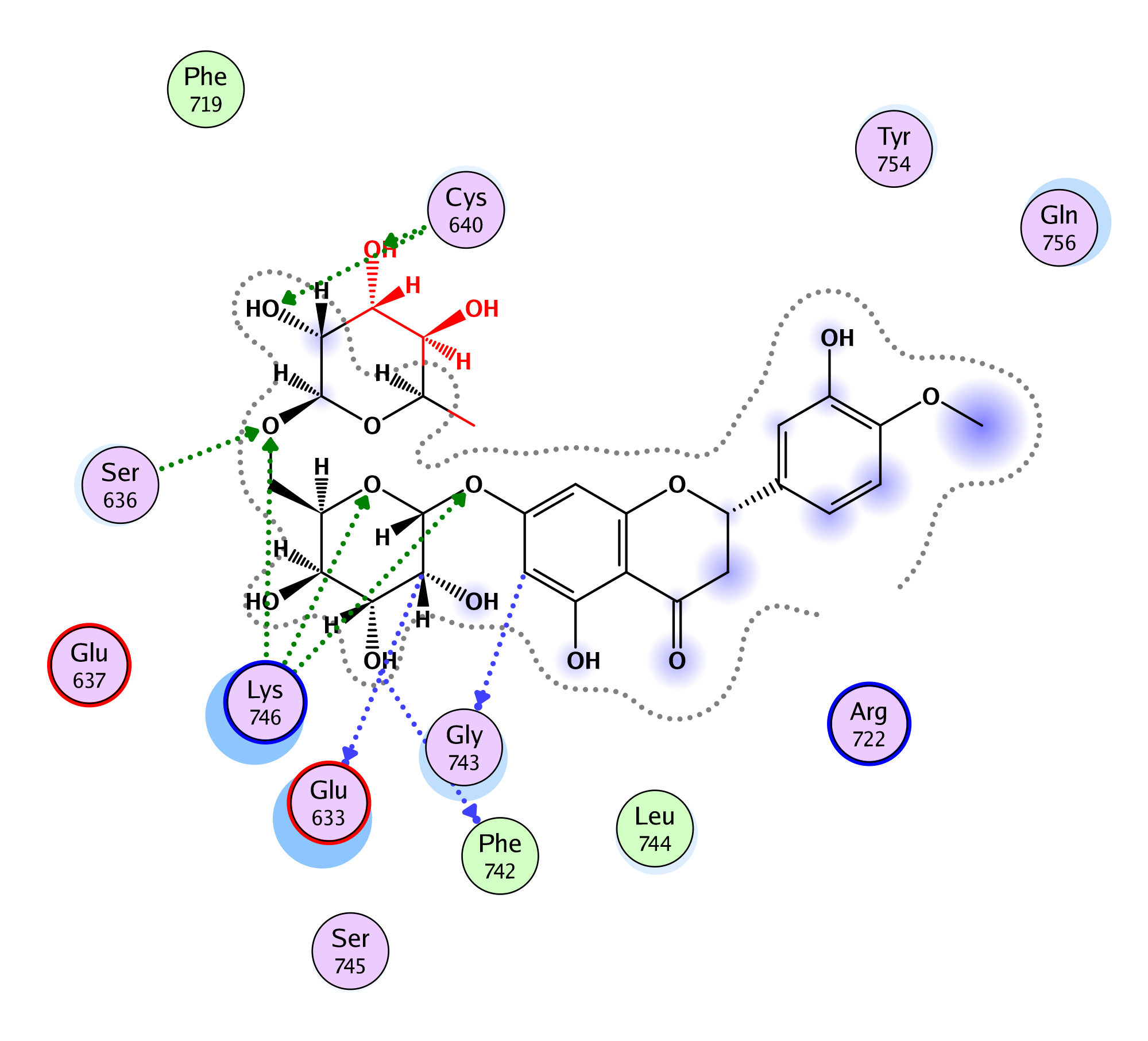

Supplement: S1 File — (ZIP) [file pone.0334106.s001.zip › figure tf formate/FIG1D.tiff]

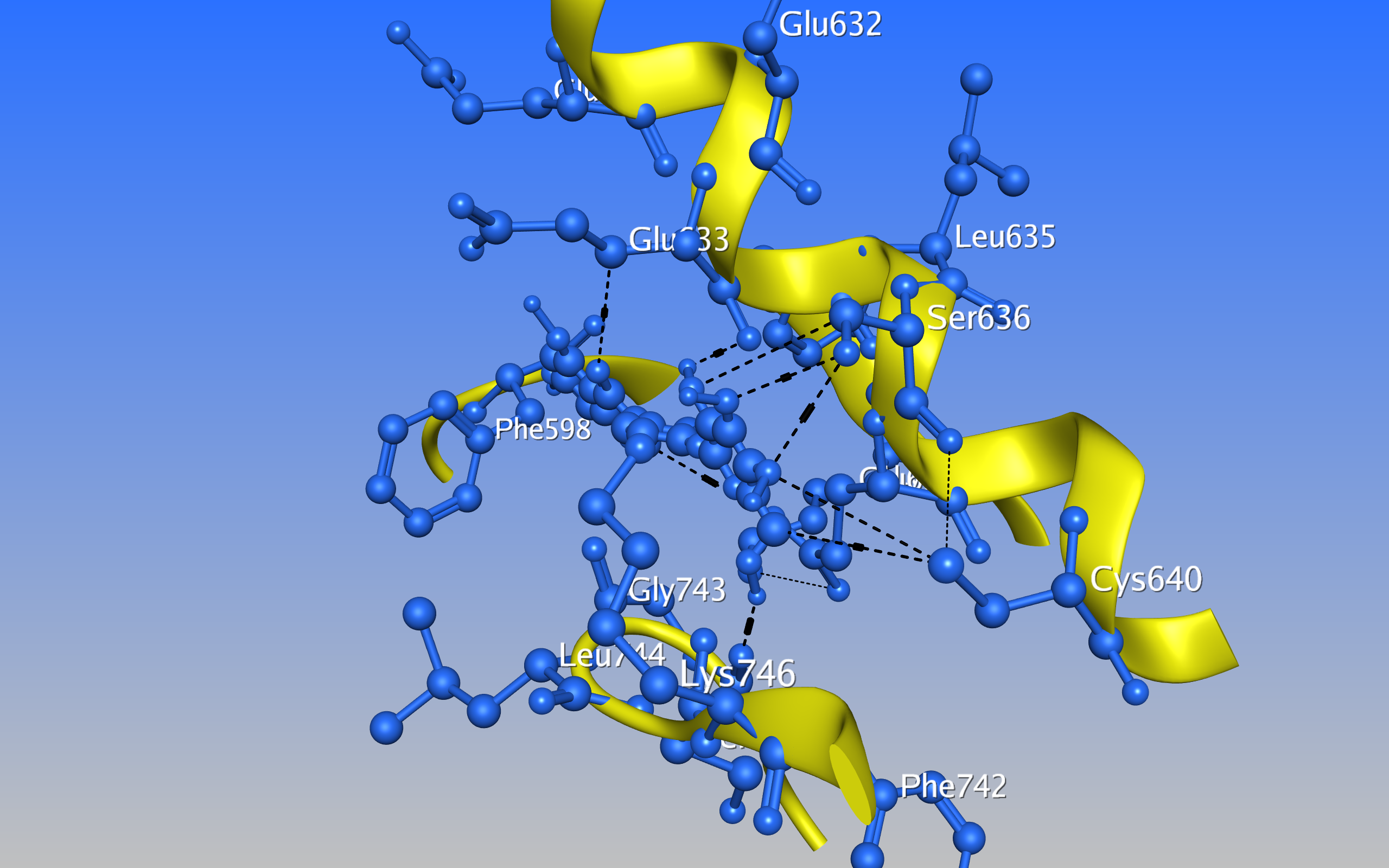

Supplement: S1 File — (ZIP) [file pone.0334106.s001.zip › figure tf formate/FIG1E.tiff]

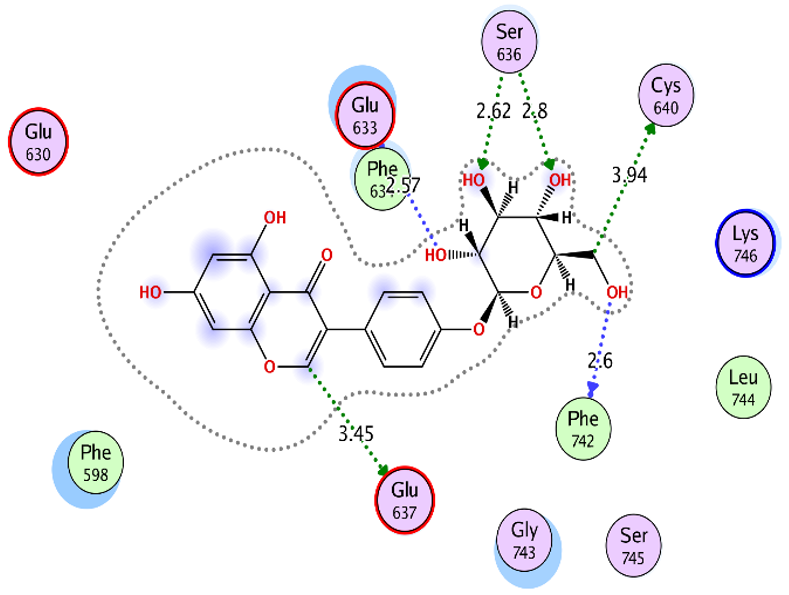

Supplement: S1 File — (ZIP) [file pone.0334106.s001.zip › figure tf formate/FIG1F.tif]

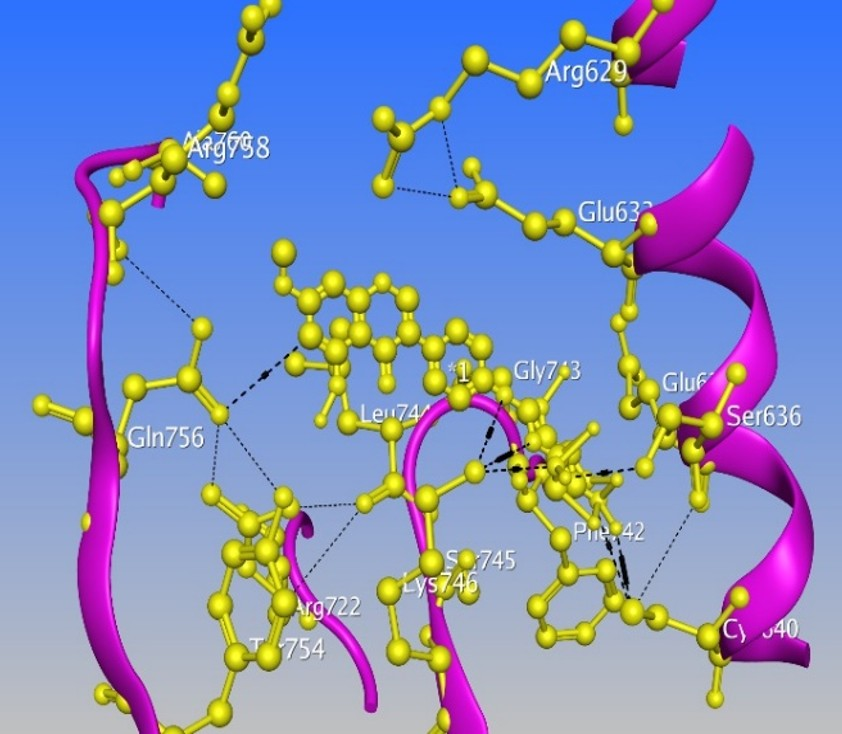

Supplement: S1 File — (ZIP) [file pone.0334106.s001.zip › figure tf formate/FIG1G.tiff]

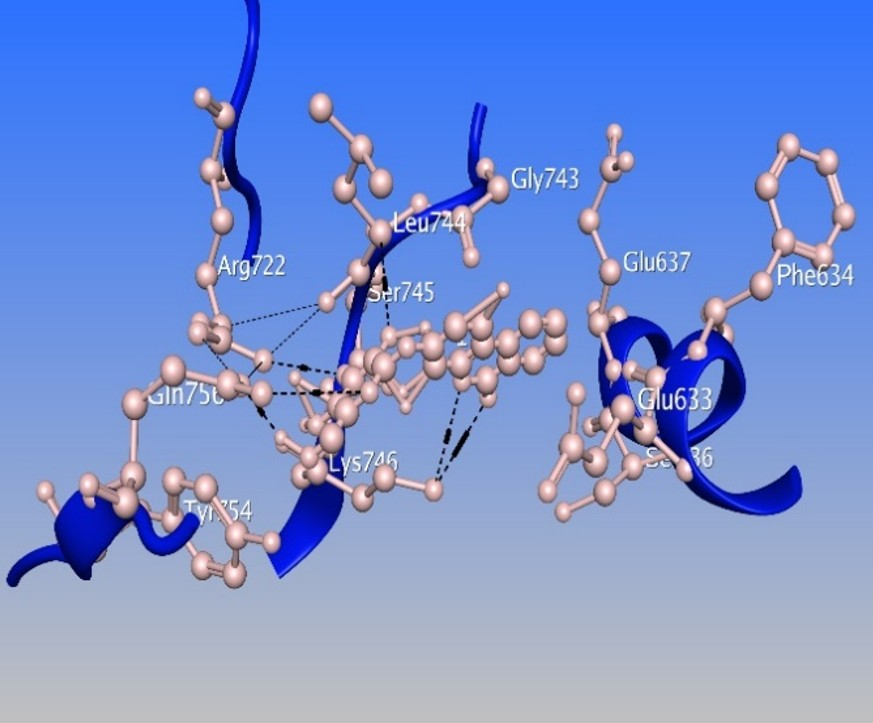

Supplement: S1 File — (ZIP) [file pone.0334106.s001.zip › figure tf formate/FIIG.1I.tiff]
